# Meiosis in budding yeast

**DOI:** 10.1093/genetics/iyad125

**Published:** 2023-08-24

**Authors:** G Valentin Börner, Andreas Hochwagen, Amy J MacQueen

**Affiliations:** Center for Gene Regulation in Health and Disease (GRHD), Department of Biological, Geological and Environmental Sciences, Cleveland State University, Cleveland, OH 44115, USA; Department of Biology, New York University, New York, NY 10003, USA; Department of Molecular Biology and Biochemistry, Wesleyan University, Middletown, CT 06459, USA

**Keywords:** YeastBook, meiosis, budding yeast, review, recombination, synaptonemal complex, chromosome segregation, cell cycle control, checkpoint

## Abstract

Meiosis is a specialized cell division program that is essential for sexual reproduction. The two meiotic divisions reduce chromosome number by half, typically generating haploid genomes that are packaged into gametes. To achieve this ploidy reduction, meiosis relies on highly unusual chromosomal processes including the pairing of homologous chromosomes, assembly of the synaptonemal complex, programmed formation of DNA breaks followed by their processing into crossovers, and the segregation of homologous chromosomes during the first meiotic division. These processes are embedded in a carefully orchestrated cell differentiation program with multiple interdependencies between DNA metabolism, chromosome morphogenesis, and waves of gene expression that together ensure the correct number of chromosomes is delivered to the next generation. Studies in the budding yeast *Saccharomyces cerevisiae* have established essentially all fundamental paradigms of meiosis-specific chromosome metabolism and have uncovered components and molecular mechanisms that underlie these conserved processes. Here, we provide an overview of all stages of meiosis in this key model system and highlight how basic mechanisms of genome stability, chromosome architecture, and cell cycle control have been adapted to achieve the unique outcome of meiosis.

## Introduction

Meiosis is the specialized cell division program used by sexually reproducing organisms to reduce their chromosome number by half, generating haploid gametes. To achieve this unique reduction in chromosome number, meiotic cells replicate their genome and then undergo two consecutive nuclear divisions without an intervening S phase. Ploidy is reduced during the meiosis I division, when homologous parental chromosomes (homologs) segregate from one another. Meiosis II is a mitosis-like division that separates sister chromatids.

Meiosis and mitosis exhibit many commonalities, prompting the idea that meiosis could be evolutionarily derived from mitosis (Wilkins and Holliday 2009). Yet, several key features of meiosis are not part of the mitotic program. When compared to mitosis, the meiotic program has evolved at least four key modifications:

(i) Homolog pairing and synapsis. As a prerequisite for their reductional segregation, homologous chromosomes physically pair during meiosis. Pairing is reinforced by the assembly of the synaptonemal complex (SC), a zipper-like structure that connects the proteinaceous axes of homologs along their entire length (Page and Hawley 2004).
(ii) Recombination. During mitosis, cohesion between sister chromatids provides a counterforce to microtubules from opposite spindle poles, thereby generating the tension needed for bipolar attachment and accurate segregation of sister chromatids (Marston 2014). During meiosis I, functionally equivalent connections between homologs are provided by inter-homolog crossovers in combination with sister-chromatid cohesion. Crossovers arise from the programmed induction, and repair, via homologous recombination, of a large number of DNA double-strand breaks (DSBs). While primarily serving a critical mechanistic function in chromosome segregation, crossovers also have an important evolutionary role because the resulting new allele combinations increase genetic diversity in offspring.
(iii) Stepwise loss of cohesion and kinetochore architecture. In mitotic cells at metaphase, the sister kinetochores of replicated chromosomes are bioriented, and inter-sister cohesion is lost along the entire length of chromosomes at the metaphase-anaphase transition. During the metaphase-anaphase transition of meiosis I, each pair of sister kinetochores is co-oriented, and cohesion along chromosome arms is selectively eliminated. Pericentromeric sister chromatid cohesion, by contrast, is protected during meiosis I, to be eliminated only during meiosis II. These modifications ensure that homologs segregate during meiosis I, whereas sister-chromatids segregate during meiosis II (Marston 2014).
(iv) Replication suppression prior to meiosis II. During meiosis, one round of replication is followed by two rounds of chromosome segregation. To achieve this unusual cell cycle pattern, replication initiation must be suppressed between meiosis I and meiosis II (Benjamin et al. 2003; Phizicky et al. 2018).

All four meiosis-specific modifications are conserved among sexually reproducing eukaryotes (Ramesh *et al.* 2005). Meiosis furthermore is typically embedded within a larger program of gametogenesis that either packages the meiotic products for fertilization or prepares them for the haploid phase of the life cycle. In *Saccharomyces cerevisiae*, meiosis is integrated with a starvation response and a developmental process that encapsulates the four gametes with stress-resistant cell walls to form a tetrad of spores inside an ascus (Neiman 2011).

The budding yeast *S. cerevisiae* has become a major model for meiosis research due to several key features. (i) Most budding yeast genes central to meiosis are conserved among sexually reproducing organisms, including animals, plants and fungi (Ramesh *et al.* 2005). (ii) Near synchronous meiosis can be induced in large cultures by simple manipulation of nutritional conditions (Börner and Cha 2015). (iii) The four haploid spore products of meiosis remain connected as a tetrad, allowing the investigator to isolate and analyze all products of a single meiosis. (iv) Spores resume haploid growth allowing ready analyses of genotypes and phenotypes. (v) Events of chromosome morphogenesis and the localization of chromosomal proteins can be observed using immunofluorescence microscopy of surface-spread or live cells (Sym *et al.* 1993; Koszul *et al.* 2008). (vi) Recombination intermediates and products can be directly monitored by physical analysis of DNA molecules containing recombination hotspots (Ahuja and Borner 2011). Importantly, these tools and features of the budding yeast experimental system allow one to assess, in the same cell population, transitions in global chromosome architecture as well as the molecular events that occur between DNA duplexes (Kim *et al.* 2010).

**Fig. 1.**
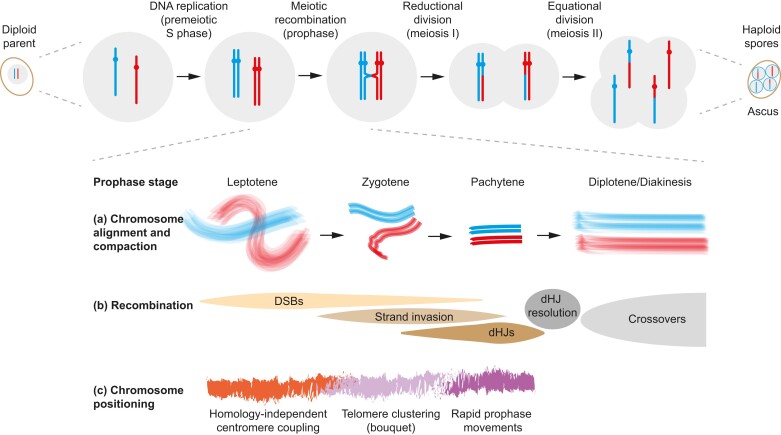
Timeline of meiosis. Top panels show changes in chromosome number and recombination as cells progress from premeiotic DNA replication, through meiotic prophase I into the two meiotic divisions. A diploid mother cell ultimately gives rise to an ascus-enclosed tetrad of four genetically distinct haploid spores. Lower panels schematically depict changes in a) chromosome alignment and compaction, b) intermediate stages of recombination, and c) chromosome positioning during the stages of meiotic prophase I.

In describing our current understanding of the molecular processes that underpin meiosis, this review will largely follow the temporal order outlined in Fig. 1 while considering causal relationships between parallel processes in DNA metabolism and chromosome morphogenesis. We place particular emphasis on meiotic prophase I, the extended cell-cycle stage when many meiosis-specific patterns are established, and the two meiotic nuclear divisions, when functional outcomes of these patterns are realized.

## Meiotic entry and pre-meiotic S phase

The meiotic program is initiated by a major wave of gene expression mediated by the master transcriptional regulator Ime1 and its co-activator Ume6 (Kassir *et al.* 1988, 2003; Mandel *et al.* 1994; Rubin-Bejerano *et al.* 1996). *IME1* activation involves a number of integrated intrinsic and extrinsic cues, including mating-type heterozygosity, low nutrient availability, and mitochondrial activity, to ensure that only respiration-competent diploid cells under severe nutrient limitation activate the meiotic program (Simchen and Kassir 1989; Treinin and Simchen 1993; Jambhekar and Amon 2008; Weidberg *et al.* 2016). Ime1/Ume6 induce starvation response genes and factors involved in pre-meiotic replication, recombination, and chromosome morphogenesis (Chu *et al.* 1998; Primig *et al.* 2000).

Several additional layers of regulation fine-tune meiotic entry. First, although rare in the yeast genome, introns can be found in multiple meiosis-specific genes (Juneau *et al.* 2007). While primary transcripts of these genes are detectable in premeiotic cells, splicing of their introns tends to be meiosis-specific and depends on the meiotic splicing activator Mer1, thereby ensuring that mature transcripts are restricted to meiosis (Engebrecht *et al.* 1991). Second, Ime1 induces the expression of numerous non-coding transcripts (Brar *et al.* 2012; Kim Guisbert *et al.* 2012). In several cases, production of these non-coding transcripts impedes the expression of overlapping genes, and thus allows Ime1 to also downregulate genes (Chen *et al.* 2017; Chia *et al.* 2017). Finally, the m^6^A methyltransferase Ime4 mediates large-scale methylation of meiotic mRNAs (Clancy *et al.* 2002; Schwartz *et al.* 2013). Methylation in the 3′ untranslated region of *IME1* mRNA counters binding of the meiotic repressor Rme1, which increases *IME1* transcript levels and locks cells into the meiotic program (Shah and Clancy 1992; Agarwala *et al.* 2012; Bushkin *et al.* 2019).

In the fast sporulating “SK1” yeast strain background, premeiotic S phase initiates within about an hour of exposing diploids to severe starvation (Cha *et al.* 2000), whereas this transition is substantially slower and less synchronous in other strain backgrounds commonly used for meiosis research, such as “BR2495” (Sym *et al.* 1993). Pre-meiotic DNA replication is similar to vegetative replication in that it uses the general replication machinery, initiates largely at the same origins, and requires Dbf4-dependent kinase (DDK) (Collins and Newlon 1994; Valentin *et al.* 2006; Mori and Shirahige 2007; Blitzblau *et al.* 2012). However, the regulation of cyclin-dependent kinase (CDK) is altered in several ways during meiosis. Unlike in vegetative cells, the initiation of pre-meiotic DNA replication depends absolutely on the S-phase cyclins Clb5 and Clb6 (Stuart and Wittenberg 1998). Moreover, the CDK-like meiotic kinase Ime2 replaces the G1-CDKs (Cdc28-Cln1-3) in mediating the proteasomal degradation of the CDK inhibitor Sic1 (Dirick *et al.* 1998; Benjamin *et al.* 2003). This independence from G1 cyclins ensures that meiotic cells do not undergo bud formation (Colomina *et al.* 1999). The low nucleotide availability under starvation conditions and concurrent initiation of changes in chromosome morphology cause replication in meiotic cells to be slower and less synchronous compared to vegetative cells (Cha *et al.* 2000; Blitzblau *et al.* 2012; Hong *et al.* 2019). Perhaps to accommodate these delays and to prevent DSBs from blocking progression of the replication fork, several mechanisms restrict recombination initiation to replicated DNA (Borde *et al.* 2000; Hochwagen *et al.* 2005; Blitzblau and Hochwagen 2013; Murakami and Keeney 2014).

## Architecture and assembly of axial elements

Coincident with their replication, meiotic chromosomes initiate a program of chromatin loop formation and compaction, which changes their microscopic appearance from an amorphous chromatin “cloud” to distinct chromosomal bodies (Zickler and Kleckner 1998, 1999). The distinctive appearance of chromosomes helps to define five substages of the ensuing meiotic prophase and is also associated with key molecular events at the DNA level (Fig. 1) (Padmore *et al.* 1991; Zickler and Kleckner 1998, 1999). During the leptotene stage, as DNA replication is completed and programmed recombination is initiated, the chromatin of DAPI-stained, surface-spread nuclei appears diffuse like frayed cotton. During zygotene, individual chromosomes thicken and become more thread-like as they develop a meiosis-specific, protein-rich “core” called the axial element. At this stage, chromosome axes begin to align in pairs as DNA breaks identify homologous regions for processing into recombination products. At pachytene, homologous chromosomes are maximally thickened around a lengthwise-aligned pair of compacted axes, exhibiting a level of individualization that far exceeds that of yeast mitotic metaphase chromosomes. Aligned pachytene homologs feature an abundance of joint molecule (JM) inter-homolog recombination intermediates. Toward the end of pachytene or in diplotene, recombination intermediates are resolved, and chromosomes progressively lose their individualization, again appearing diffuse (J. S. Ahuja and G.V.B., unpublished) (Padmore *et al.* 1991; Klein *et al.* 1999; Zickler and Kleckner 1999).

**Fig. 2.**
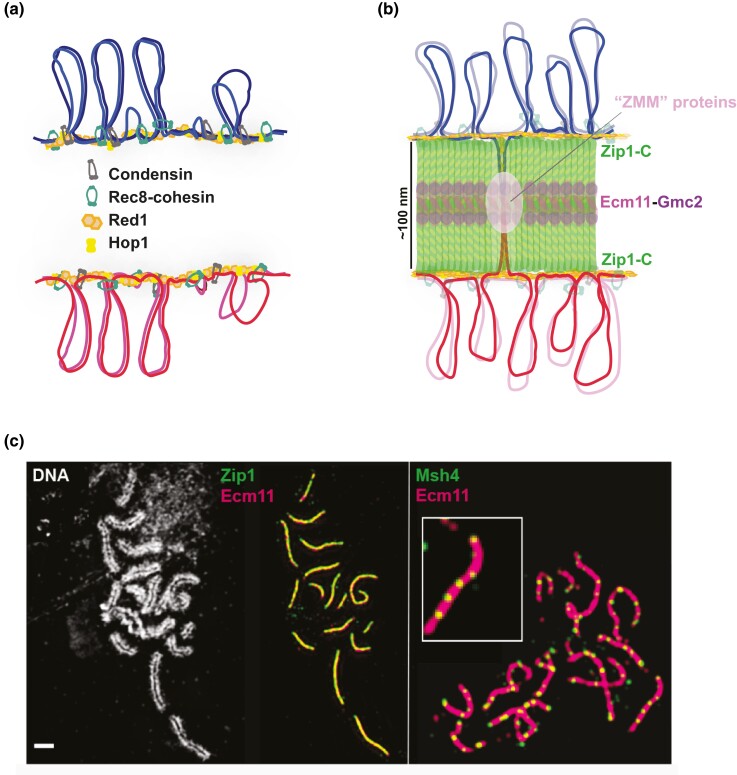
Meiotic chromosome axis and SC development. a) Illustration of meiotic chromosome axis development. SMC ring complexes (condensin, grey; meiotic cohesin, green) promote the formation of ∼20 kb chromatin loops, through embrace of discrete, non-contiguous regions of a single DNA molecule, and/or loop extrusion activity. Sister chromatid loops anchor to a shared, protein-rich axis. Red1 and Hop1 proteins (orange, yellow) localize along the length of chromosome axes during leptotene and promote the formation of Spo11-mediated DNA double strand breaks, initiating homologous recombination and pairing between homologous chromosomes. b) Schematic of synaptonemal complex (SC) in budding yeast, which generates a ∼100 nm bridge between axes along the length of partner chromosomes. SC assembly involves the multimerization of several proteins, including the transverse filament protein Zip1 and central element proteins Ecm11 and Gmc2. Zip1 forms parallel dimers through an extended central coiled-coil region; two Zip1 dimers span the width of the SC with their C termini alongside chromosome axes and their N termini toward the SC midline. The Ecm11–Gmc2 complexes assemble at the midline of the SC. A subset of interhomolog recombination intermediates is processed by ZMM proteins (pink circle) into stable joint molecules (e.g. double Holliday junctions). SC assembly depends upon recombination initiation and ZMM proteins, and initiates from such nascent crossover-fated recombination sites. c) Surface-spread, immunostained chromosomes from *S. cerevisiae* mid-meiotic prophase nuclei, imaged using structured illumination microscopy. Left and middle panel: SC proteins (Zip1-N termini, green; Ecm11, magenta) are observed at the interface of aligned homologous chromosome axes (DAPI-stained DNA, white). Right panel: The ZMM protein Msh4 (green, far right panel) marks interhomolog crossover-designated recombination intermediates associated with the central element (Ecm11, magenta) of the SC. Bar, 1 μm.

In ultrastructural images, zygotene and pachytene chromosomes appear as linear arrays of chromatin loops, each array anchored to a protein-rich axis (Fig. 2, a and c) (Moens and Pearlman 1988; Zickler and Kleckner 1999). Chromatin loops have an estimated average size of 20 kb and their formation depends on the meiosis-specific cohesin complex in which Rec8 replaces the canonical kleisin Scc1 (a.k.a. Mcd1) (Klein *et al.* 1999; Muller *et al.* 2018; Schalbetter *et al.* 2019). Consistent with a foundational role for Rec8-cohesin in axial element formation, Rec8 binding sites coincide with chromatin loop boundaries identified in Hi-C experiments, suggesting that cohesin localizes at the base of chromatin loops (Muller *et al.* 2018; Schalbetter *et al.* 2019). Rec8-cohesin is preferentially enriched between convergent gene pairs, resulting in a quasi-regular binding pattern along the length of meiotic chromosomes (Glynn *et al.* 2004; Sun *et al.* 2015). Although looping patterns appear reproducible at a population level, modeling and experimental data indicate that Rec8-cohesin occupancy is variable between cells and even from chromatid to chromatid within a pair of homologs, possibly contributing to cell-specific usage of recombination sites (Schalbetter *et al.* 2019).

Analogous to the mechanism of cohesion in mitotically dividing cells, the meiotic cohesin complex associates with chromatin prior to premeiotic replication. Upon passage of the premeiotic replication fork, cohesin becomes cohesive through acetylation of the universal cohesin component Smc3 by acetyltransferase Eco1 (Marston 2014). How Rec8-cohesin promotes the formation of arrayed chromatin loops remains unclear, but this process may involve chromatin loop extrusion. Rec8-cohesin contains ATP-dependent DNA motors of the SMC-family, which in several related complexes can promote the extrusion of DNA, resulting in the formation of loops (Terakawa *et al.* 2017; Ganji *et al.* 2018; Davidson *et al.* 2019). Indeed, meiotic cells depleted for Pds5, a negative regulator of cohesin ATPase activity, display strikingly shortened axial elements. As shorter axes are expected to correspond to longer loops, this phenotype supports the importance of cohesin's DNA extrusion activity in the formation of chromatin loop-arrays (Jin *et al.* 2009; Petela *et al.* 2018; Song *et al.* 2021). In addition, another SMC complex, condensin, localizes to axial elements and imparts a certain degree of axial compaction, as well as chromosome individualization (Yu and Koshland 2003; Zhang *et al.* 2014).

When Rec8's vegetative paralog Scc1 is ectopically expressed during meiosis as the sole kleisin subunit, it fails to support proper recombination and SC assembly even though it localizes to the same chromosomal sites as Rec8 and promotes sister chromatid cohesion (Toth *et al.* 2000; Lee and Amon 2003; Brar *et al.* 2009; Sun *et al.* 2015). This indicates that Rec8 serves unique roles during meiosis beyond sister cohesion and loop extrusion. Such roles likely include the recruitment of the meiosis-specific axis proteins: Red1 and Hop1 are major regulators of meiotic recombination that localize to axial element structures in a manner that is largely dependent on Rec8-cohesin and possibly condensin (Hollingsworth *et al.* 1990; Smith and Roeder 1997; Klein *et al.* 1999; Yu and Koshland 2003; Panizza *et al.* 2011). Red1 and Hop1 do not share the foundational structural role of Rec8-cohesin in axis assembly because Rec8-cohesin localizes normally along chromosomes in the absence of Red1 (Sun *et al.* 2015). Moreover, proteinaceous chromosomal core structures detected by electron microscopy are morphologically intact in both *red1* and *hop1* mutants, suggesting the formation of at least a nascent axial element structure (Rockmill and Roeder 1990; Klein *et al.* 1999). At the same time, *hop1* mutants display genome-wide shifts in loop structure (Schalbetter *et al.* 2019) and loss of either Red1 or Hop1 results in abnormally diffuse mid-meiotic prophase chromosome morphology, indicating a role for these proteins in chromosome compaction (Nag *et al.* 1995; Yu and Koshland 2003). Red1 and Hop1 may affect higher-order chromatin folding via their critical functions in meiotic DSB formation, as a DSB-deficient *spo11* mutant displays chromosome individualization defects similar to *red1* and *hop1* mutants (Mao-Draayer *et al.* 1996; Smith and Roeder 1997; Klein *et al.* 1999; Macqueen and Roeder 2009; Yisehak and MacQueen 2018).

Recruitment of Red1 to chromosome axes likely depends on its interaction with Rec8, as suggested by co-immunoprecipitation as well as proximity labeling experiments (Sun *et al.* 2015). Hop1, in turn, binds to Red1 (De Los Santos and Hollingsworth 1999; Woltering *et al.* 2000; West *et al.* 2018) and depends on Red1 for its association with Rec8-associated axial elements (Smith and Roeder 1997; Sun *et al.* 2015). In addition, Red1 and Hop1 also bind independently of Rec8 in regions with elevated nucleosome density and dependent on Hop1's PHD-like domain (Heldrich *et al.* 2022). Finally, Hop1 and Red1 also exhibit in vitro DNA binding activity with a preference for non-duplex, branched DNA, raising the possibility that the axis-association of Hop1 and Red1 involves direct engagement with DNA (Kironmai *et al.* 1998; Kshirsagar *et al.* 2017). Both Red1 and Hop1 can form higher-order assemblies: Red1 forms homo-tetrameric bundles that can further oligomerize (Woltering *et al.* 2000; West *et al.* 2019), while the HORMA (Hop1-Rev7-Mad2) domain of Hop1 binds to so-called “closure” protein-protein interaction motifs in Red1's C terminus (West *et al.* 2018). Hop1 also binds a closure motif in its own C-terminus (West *et al.* 2018), potentially allowing for the formation of higher-order Hop1 assemblies, as demonstrated for several Hop1 orthologs in *C. elegans* (Kim *et al.* 2014).

Somewhat surprisingly, axial element assembly occurs independent of DNA replication even though the two processes normally happen contemporaneously. Double mutants missing the cyclins Clb5 and Clb6 fail to initiate pre-meiotic DNA synthesis but show normal enrichment patterns for Rec8, Red1, and Hop1 (Smith *et al.* 2001; Blitzblau *et al.* 2012). Furthermore, Red1 and Hop1 assemblies formed in the absence of replication support proper axial element function, as chromosomes in replication-deficient *cdc6-mn* (meiotic null) mutants undergo homolog pairing, at least some SC assembly, and (interhomolog) recombination (Hochwagen *et al.* 2005; Brar *et al.* 2009; Blitzblau *et al.* 2012). Thus, meiotic cohesin mediates axis assembly even when it does not provide cohesion between sister chromatids.

## Meiotic recombination

### DSB formation

DSB formation and processing are an integral part of the meiotic program. Recombination is initiated by the formation of ∼170 DSBs in every meiotic nucleus, distributed along most of the yeast genome (Nicolas *et al.* 1989; Padmore *et al.* 1991; Pan *et al.* 2011). Genome-wide analyses have identified ∼3,600 meiotic DSB hotspots, a subset of which is used in different cells within a population (Gerton *et al.* 2000; Blitzblau *et al.* 2007; Buhler *et al.* 2007; Pan *et al.* 2011). DSBs form in a largely sequence non-specific manner and occur primarily in nucleosome-free promoter regions, within segments of 200–1,000 bp. At the most active hotspots, DSB formation is sufficiently common to be detectable by Southern blot analysis, with the engineered *HIS4::LEU2* hotspot breaking in essentially every cell (Cao *et al.* 1990; Zhang *et al.* 2011).

**Fig. 3.**
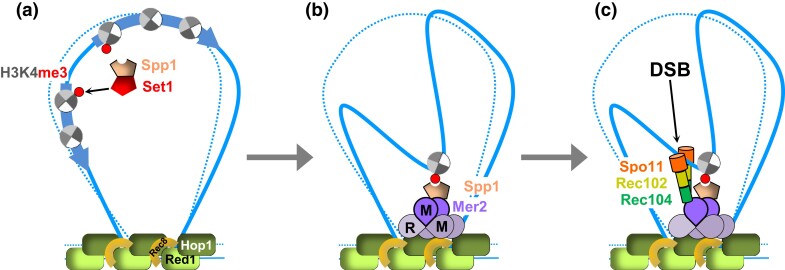
Tethered loop-axis model of meiotic DSB formation. a) DSBs occur in chromatin loops devoid of axis proteins Red1, Hop1 and cohesin kleisin subunit Rec8, in nucleosome-free regions upstream of transcription start sites or between diverging transcription units (arrows). Sectored circles indicate nucleosomes, with histone H3 of the first histone of each transcription unit carrying a trimethylation at lysine K4. H3K4 trimethylation and H3K4 recognition are carried out by COMPASS complex components Set1 and Spp1, respectively. b) Loop DNA is recruited to the emerging chromosome axis via interaction between histone reader Spp1 with both H3K4^me3^ and the axis-associated RMM complex. c) The RMM complex recruits the catalytic core comprising Spo11–Rec102–Rec104 together with Ski8 (not shown) to initiate DSB formation in loop sequences, but in association with chromosome axes. The DSB formation and resection MRX complex also localizes to DSB sites but is omitted for clarity.

DSB formation depends on three physically interconnected yet functionally distinct protein subcomplexes that together control the catalytic activity of the DSB-forming enzyme Spo11 (Fig. 3). Spo11 is a meiosis-specific transesterase that shares sequence similarity with the catalytic component of archaebacterial topoisomerase VI (Bergerat *et al.* 1997). Like other type II topoisomerases, two Spo11 molecules undergo nucleophilic attack of phosphates in both DNA strands via a highly conserved tyrosine, generating 2-nucleotide staggered 5′ overhangs at the cleaved site (De Massy *et al.* 1995; Liu *et al.* 1995; Xu and Kleckner 1995; Keeney *et al.* 1997; Claeys Bouuaert *et al.* 2021). Unlike topoisomerase VI, however, Spo11 does not re-ligate the cleaved strands, but remains covalently attached to the DNA ends, producing a protein-capped DSB (Keeney *et al.* 1997).

Archeal topoisomerase VI is a heterotetramer comprising two A and two B subunits (Forterre *et al.* 2007). The A subunit is characterized by the “Toprim” domain also found in Spo11 (and other topoisomerases and primases), whereas Spo11 interaction partners Rec102 and Rec104 jointly exhibit a remote similarity with the B subunit of the type-II topoisomerase gate complex (Salem *et al.* 1999; Robert *et al.* 2016; Vrielynck *et al.* 2016; Claeys Bouuaert *et al.* 2021). Ski8 as the fourth protein in the catalytic DSB core complex lacks sequence similarity with topoisomerase VI but interacts with Spo11 directly as a presumed scaffolding component (Arora *et al.* 2004; Claeys Bouuaert *et al.* 2021). Whereas Spo11, Rec102, and Rec104 are meiosis-specific proteins, Ski8 also performs functions unrelated to recombination during vegetative growth in the cytoplasm but translocates to the nucleus during meiosis (Arora *et al.* 2004).

Spo11 activity requires two additional subcomplexes that couple DSB formation to the axial element and to DSB processing, respectively, thereby ensuring that Spo11-DSBs only form when they can readily be repaired. The meiosis-specific Rec114–Mer2–Mei4 (RMM) complex consists of Rec114, Mer2 (a.k.a. Rec107), and Mei4 (Fig. 3b). Mer2, which is capable of forming a phase-separated condensate, interacts with Rec102/Rec104, assembles DNA-mediated nucleoprotein ensembles, and recruits Spo11 complexes (Menees and Roeder 1989; Malone *et al.* 1991; Rockmill, Engebrecht, *et al.* 1995; Li *et al.* 2006; Maleki *et al.* 2007; Claeys Bouuaert *et al.* 2021). Mer2 further couples DSB formation to replication, undergoing consecutive phosphorylation by CDK and DDK (Henderson *et al.* 2006; Sasanuma *et al.* 2008; Wan *et al.* 2008). Mer2 phosphorylation by replisome-associated kinase DDK ensures that DSBs form only after passage of the replication fork, although this coupling appears to be bypassed in *cdc6-mn* mutants (Blitzblau and Hochwagen 2013; Murakami and Keeney 2014).

A third protein subcomplex required for meiotic DSB formation, MRX, is shared with vegetative DNA damage response (DDR) pathways. MRX comprises the endo/exonuclease Mre11, Rad50, an SMC protein and ATPase that assembles into a large ring structure capable of embracing and/or bridging DNA molecules, as well as Xrs2, a protein required for the nuclear translocation of Mre11 and Rad50 (Oh *et al.* 2016). Whereas the role of MRX in vegetative cells is limited to DSB resection, during meiosis it is also indispensable for DSB formation. Like Spo11, chromosomal distribution of MRX is strongly correlated with DSB positions and frequencies (Borde *et al.* 2004; Pan *et al.* 2011). Functions of MRX in DSB formation and resection are separable, as *mre11S* and *rad50S* alleles are functional for DSB formation yet defective for resection (below) (Alani *et al.* 1990; Nairz and Klein 1997).

Unlike other Spo11-interacting proteins, the RMM complex is not enriched at DSB hotspots, but instead localizes to axial-element sites likely via interaction with Hop1 (Panizza *et al.* 2011). The positional anticorrelation between DSB sites and axis protein Red1 as well as the RMM complex led to the “tethered-loop-axis complex” model, where Spo11 cuts DSB sites located in non-axis associated loop DNA, thereby bringing the recombination site to the chromosome axis for subsequent processing steps (Fig. 3) (Blat *et al.* 2002; Panizza *et al.* 2011; Acquaviva *et al.* 2013; Sommermeyer *et al.* 2013). Notably, however, DSBs are less abundant but not abolished in mutants missing axial element proteins Rec8 or Red1, indicating that RMM complexes can activate DSB formation without an axial element (Mao-Draayer *et al.* 1996; Schwacha and Kleckner 1997; Blat and Kleckner 1999; Klein *et al.* 1999; Carballo *et al.* 2008; Kugou *et al.* 2009; Sun *et al.* 2015).

The exact position of DSBs within chromatin loops is controlled epigenetically, via interaction of the DSB machinery with trimethylated histone H3 lysine 4 (H3K4me3). This histone modification occurs predominantly at the first nucleosome within ORFs, both in meiotic and vegetative cells, accounting for frequent association of DSB hotspots with (divergent) promoters (Sollier *et al.* 2004; Blitzblau *et al.* 2007). Interaction between meiotic axes and loop-located H3K4me3 occurs via the RMM component Mer2, which interacts with the histone modifying COMPASS complex to form a physical bridge between DSB site and axial element (Pan *et al.* 2011; Acquaviva *et al.* 2013; Sommermeyer *et al.* 2013). The COMPASS complex carries out trimethylation of histone H3K4 via its catalytic component Set1 (Fig. 3a). Links between DSB sites and the RMM complex are directly stabilized by another COMPASS complex component, the histone reader Spp1, which can simultaneously interact with H3K4me3 via its PHD finger motif and with Mer2 (Acquaviva *et al.* 2013; Sommermeyer *et al.* 2013; Rousova *et al.* 2021). Importantly, targeting Spp1 to a region that lacks H3K4me3 suffices to induce Spo11-mediated DSB formation (Sommermeyer *et al.* 2013). H3K4 trimethylation further depends on mono-ubiquitylation of histone H2B by the E2/E3 ubiquitin ligase pair Rad6/Bre1 and on the PAF1C complex, explaining effects of these proteins on DSB formation (Sollier *et al.* 2004; Yamashita *et al.* 2004; Gothwal *et al*. 2016). At the same time, not all H3K4me3 sites are correlated with DSBs and vice versa, suggesting the involvement of other determinants (Borde *et al.* 2009; Bani Ismail *et al.* 2014).

Finally, the 26S proteasome is recruited to chromosomes in a meiosis-specific manner at the time of DSB formation and is required for efficient DSB formation, raising the possibility that DSB formation involves protein degradation in close proximity to the chromosome axis (Ahuja *et al.* 2017; Yang *et al.* 2022).

### DSB resection

**Fig. 4.**
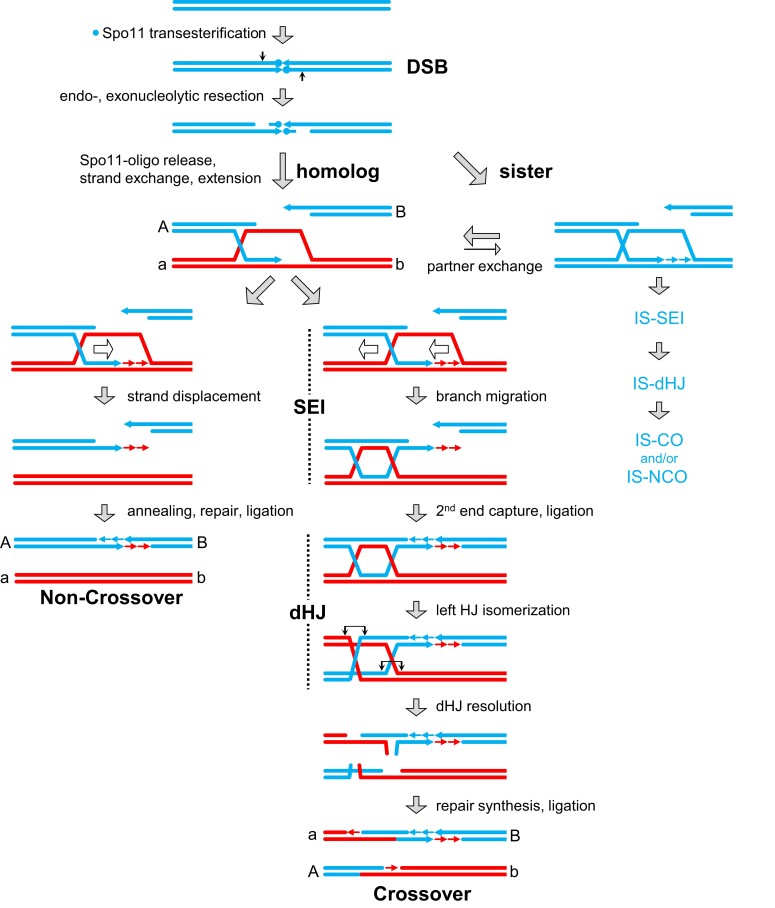
Homologous recombination pathways during meiosis. The recombination model shows two allelic double stranded DNA molecules from homologous chromosomes in blue and red. Spo11, covalently attached to the 5′ ends of the cleaved strand, is indicated by a filled circle. 3′ ends and newly synthesized DNA are indicated by arrowheads and short arrows in blue or red, respectively, without consideration of ligation status. Recombination between sister chromatids likely involves equivalent intermediates as recombination between homologs, and only the names of the relevant molecules are provided (intersister single end invasion, IS-SEI; intersister double Holliday junction, IS-dHJ). Single end invasion (SEI) and double Holliday junction (dHJ), molecules are thought to exist in two conformations, as indicated by the dotted vertical line in black. Movements of Holliday junctions are indicated by open arrows. To generate crossovers from dHJs, the two tandem Holliday junctions need to be resolved with opposite directionalities, either by two single stranded nicks that flank each junction or via nicks of both crossing strands (see text for details).

DSB formation is rapidly followed by 5′ resection, during which both Spo11-capped DNA strands are nicked endonucleolytically up to 300 nucleotides from the DSB site via the single-stranded nicking activities of the MRX complex in association with Mre11 activator Sae2 (a.k.a. Com1) (Fig. 4) (Neale *et al.* 2005; Garcia *et al.* 2011; Cannavo and Cejka 2014; Anand *et al.* 2016; Arora *et al.* 2017). Accordingly, unresected DSBs physically linked to Spo11 accumulate in *sae2D* [*delta*] as well as *mre11S* and *rad50S* meiotic cells. (Alani *et al.* 1990; Keeney *et al.* 1997; McKee and Kleckner 1997a; Nairz and Klein 1997; Prinz *et al.* 1997). Resection initiation depends on Sae2 phosphorylation by checkpoint kinases Tel1^ATM^ and Mec1^ATR^, with the former playing a more critical role in resection during early meiosis when DSB abundance is low (Cartagena-Lirola *et al.* 2006; Joshi *et al.* 2015; Mimitou *et al.* 2017). Using the nick as an entry point for exonucleolytic resection, the MRX complex resects towards the DSB site in the 3′ to 5′ direction, releasing two Spo11-linked oligonucleotide species 23-37 and <12 nucleotides in length (Neale *et al.* 2005; Garcia *et al.* 2011). Exonuclease Exo1 resects away from the DSB site, extending the single stranded resection tract to ∼800 nucleotides (Tsubouchi and Ogawa 2000; Zakharyevich *et al.* 2010; Mimitou *et al.* 2017). The resulting 3′ single-stranded overhangs form the substrate for all subsequent homology search and strand exchange reactions.

### DSB strand exchange and recombination pathway choice

To initiate homology-directed DSB repair, the single-stranded 3′ DNA overhang invades an intact double-stranded DNA template, either on the homologous chromosome (red; Fig. 4) or on the sister chromatid (blue), displacing an intact DNA strand with the same directionality and undergoing base pairing with the complementary strand giving rise to a D-loop intermediate. All interhomolog recombination events likely are initiated by nascent D-loops involving side-by-side (paranemic) interactions between single-stranded DNA segments, which are subsequently converted into a topologically interwound (plectonemic) interaction (Hunter and Kleckner 2001). A subset of these early intermediates eventually progresses into stable interhomolog single-end invasions (SEIs), the earliest detectable JM recombination intermediates associated with the crossover outcome (Allers and Lichten 2001a; Hunter and Kleckner 2001). By contrast, D-loop intermediates that give rise to non-crossovers have eluded detection by 2D gel Southern blot analysis likely because they are unstable (Börner *et al.* 2004). Single end invasions are converted into interhomolog double-Holliday junctions following DNA synthesis at both invading 3′ ends, capture of the second DSB end, and re-ligation of DSBs (Schwacha and Kleckner 1995; Lao *et al.* 2008). Double-Holliday junctions entail fully-ligated DNA strands that are separated by ∼260 bp of heteroduplex DNA between the two junctions, corresponding to ∼90 nm of B-form duplex DNA (Bell and Byers 1983; Schwacha and Kleckner 1995; Cromie *et al.* 2006; Oh *et al.* 2008). Prior to DSB second end capture, the Holliday junction frequently branch migrates away from the DSB site, generating a double-Holliday junction positioned entirely on one side of the DSB (Allers and Lichten 2001b; Lao *et al.* 2008; Ahuja *et al.* 2021) (Fig. 4). Consistent with this model, heteroduplex DNA in crossover products is often detected only on one side of the DSB (Allers and Lichten 2001b; Ahuja *et al.* 2021).

Whereas DSB repair in mitotically dividing cells uses the sister chromatid as template (Kadyk and Hartwell 1992; Symington *et al.* 2014), during meiosis a non-sister chromatid belonging to the homolog is the preferred recombination partner, as the goal is to create crossover linkages that support homolog segregation (Schwacha and Kleckner 1997). Recombination intermediates between sister chromatids are formed at lower frequencies and are actively suppressed during meiosis (Schwacha and Kleckner 1997; Kim *et al.* 2010; Lao *et al.* 2013; Callender *et al.* 2016), although prior to stabilization of inter-homolog SEIs, invading 3′ ssDNA ends frequently change recombination templates between homolog and sister chromatid (McMahill *et al.* 2007; Marsolier-Kergoat *et al.* 2018; Sandhu *et al.* 2020; Ahuja *et al.* 2021). It is also noteworthy that intersister repair is suppressed only transiently during meiosis, as indicated by frequent repair with the sister chromatid during early meiosis (Joshi *et al.* 2015), in absence of a matching DNA sequence on the homolog (Goldfarb and Lichten 2010) and during arrest in mid-to-late prophase I (Subramanian *et al.* 2016).

Like the crossover pathway between homologs, DSB repair between sister chromatids involves intersister SEIs and presumed intersister double-Holliday junctions (Schwacha and Kleckner 1997; Kim *et al.* 2010), although it has not been ruled out that single Holliday junctions are also formed (Fig. 4). Notably, single-Holliday junctions are the predominant recombination intermediate detectable in *Schizosaccharomyces pombe* (Cromie *et al.* 2006). They are also detectable by electron microscopy in *S. cerevisiae* and may contribute to intersister recombination but alternatively may represent JM resolution intermediates (Oh *et al.* 2008).

### Roles of Dmc1 and Rad51 in DSB strand exchange

Strand exchange of the first DSB end with an intact template DNA is mediated by Dmc1 and Rad51, two orthologs of prokaryotic RecA recombinase (Bishop *et al.* 1992; Shinohara *et al.* 1992). Whereas Rad51 is also involved in homologous recombination in vegetative cells, Dmc1 is specifically expressed during meiotic prophase. Following 5′ resection, Rad51 and Dmc1 form a nucleoprotein filament at 3′ ssDNA overhangs, replacing the single-strand binding protein RPA (Gasior *et al.* 1998; Shinohara *et al.* 1998; Plate *et al.* 2008). RPA replacement is promoted by a homoheptameric ring of Rad52, although functional Dmc1 filaments can also assemble without Rad52 (Gasior *et al.* 1998, 2001; Lao *et al.* 2008). The two recombinases form separate domains in the nucleoprotein filament; Dmc1 binds to the very 3′ end of the DSB, while Rad51 localizes to the region of the single-stranded tail closest to duplex DNA (Shinohara *et al.* 2000; Brown *et al.* 2015; Crickard *et al.* 2018; Lan *et al.* 2020). Rad51 self-assembles via homotypic interactions, but also recruits Dmc1 into the filament (Shinohara *et al.* 2000; Brown *et al.* 2015; Crickard *et al.* 2018; Lan *et al.* 2020). Although both Rad51 and Dmc1 are present at meiotic DSBs, the bulk of strand exchange catalysis is carried out by Dmc1 (Bishop *et al.* 1992, Bishop 1994; Cloud *et al.* 2012). As a result, recombination occurs normally when Rad51's strand exchange activity is disrupted, whereas mutants lacking Dmc1 accumulate resected DSBs, though in some strain backgrounds, DSBs are eventually repaired with frequent use of the homolog as template (Rockmill and Roeder 1994; Cloud *et al.* 2012).

Why does meiosis in yeast and many other eukaryotes depend on two strand-exchange proteins with apparently overlapping features? For one, Dmc1 appears better suited for strand exchange in the presence of mismatches, which is a fundamental aspect of interhomolog recombination (Callender *et al.* 2016; Steinfeld *et al.* 2019). The combined presence of Rad51 and Dmc1 further ensures that the homolog rather than the sister chromatid is used as recombination partner, as indicated by frequent repair with the sister chromatid in absence of either RecA paralog (Schwacha and Kleckner 1997; Lao *et al.* 2013; Callender *et al.* 2016).

Proper function of Dmc1 and Rad51 during meiosis depends on many auxiliary factors. Mutants lacking these factors resemble *dmc1* or *rad51* deletion mutants, accumulating hyper-resected DSBs or undergoing strand exchange with the sister chromatid instead of the homolog, respectively (McKee and Kleckner 1997b; Schwacha and Kleckner 1997; Leu *et al.* 1998; Hong *et al.* 2013). Assembly of the Dmc1 nucleoprotein filament depends on the heterodimeric Mei5–Sae3 complex, (Ferrari *et al.* 2009; Chan *et al.* 2019), whereas heterodimeric Hop2–Mnd1 mediates strand exchange by providing a bridge between the Dmc1-nucleoprotein filament on the invading strand and the template duplex DNA (Tsubouchi and Roeder 2003; Kang *et al.* 2015; Crickard *et al.* 2019).

Rad51 accessory proteins perform functions analogous to those in vegetative cells [reviewed in (Symington *et al.* 2014)]. The low abundance Rad51-paralogs Rad55 and Rad57 recruit or stabilize Rad51 during initiation of nucleofilament assembly, in part by countering the Rad51-removing activity of DNA helicase Srs2 (Schwacha and Kleckner 1997; Gasior *et al.* 1998; Liu *et al.* 2011). The hetero-tetrameric Shu complex, which is composed of two additional Rad51 paralogs (Psy3 and Csm2) as well as Shu1 and Shu2, is also involved in loading and/or stabilizing the Rad51 filament (Hong *et al.* 2013; Sasanuma *et al.* 2013).

Functionality of Dmc1 and Rad51 is further modulated by two paralogous DNA translocases that play roles not only in nucleoprotein filament assembly, but also in Rad51/Dmc1 removal following strand exchange. Rad54 and Tid1 (a.k.a. Rdh54) interact with Rad51 and Dmc1, respectively, and appear to perform partially overlapping functions in the formation and/or stabilization of D-loops (Dresser *et al.* 1997; Shinohara *et al.* 1997; Nimonkar *et al.* 2012). Notably, whereas Dmc1 is meiosis-specific, Tid1 is not, suggesting that it plays additional roles in DSB repair not connected to Dmc1 (Shah *et al.* 2020). Subsequent to D-loop formation, Rad54 and/or Tid1 also displace the RecA recombinases from ssDNA at recombination sites, possibly to provide a naked ssDNA strand capable of capturing the second DSB end, and allowing access for a DNA polymerase to perform repair synthesis (Fig. 4) (Li and Heyer 2009; Wright and Heyer 2014).

In addition to their functions in displacing RecA recombinases at DSB sites, Rad54 and Tid1 also remove the respective recombinases from intact double stranded DNA that lack DSBs, thereby preventing the formation of potentially toxic recombination intermediates (Holzen *et al.* 2006; Shah *et al.* 2010; Reitz *et al.* 2021). Thus, while Rad51 and Dmc1 normally colocalize at DSB sites, each recombinase also associates with additional sites along the genome that have not undergone DSB formation when Rad54 and/or Tid1 are absent (Shinohara *et al.* 2000).

### Suppression of recombination with the sister chromatid

Although Rad51's strand exchange activity is largely dispensable for recombination during wild-type meiosis, the protein is critical for directing Dmc1-mediated strand exchange to the homologous chromosome. When Rad51 is absent, not properly incorporated into nucleoprotein filaments or aberrantly degraded, strand exchange mediated by Dmc1 alone occurs preferentially with the sister chromatid (Schwacha and Kleckner 1997; Cloud *et al.* 2012; Hong *et al.* 2013; Woo *et al.* 2020). Rad51 must also be prevented from carrying out Dmc1-independent strand exchange which generates mostly inviable gametes due to increased intersister repair and/or insufficient interhomolog crossover formation (Rockmill and Roeder 1994; Lao *et al.* 2013; Callender *et al.* 2016). Rad51 inhibition is achieved via at least two mechanisms, both of which destabilize Rad51's interaction with its activator Rad54: First, Rad54 is outcompeted for binding to Rad51 by the small, meiosis-specific protein Hed1 (Tsubouchi and Roeder 2006; Busygina *et al.* 2008). Second, the Hop1-associated kinase Mek1 phosphorylates Rad54 to destabilize its interaction with Rad51, but not with Dmc1 (Niu *et al.* 2009; Ziesel *et al.* 2022). In addition, Hed1 is stabilized via phosphorylation by Mek1, again minimizing intersister recombination (Callender *et al.* 2016).

Mek1 appears to attenuate all DSB strand exchange but suppresses intersister exchange more effectively than interhomolog exchange perhaps because its inhibitory signaling remains associated with the chromosome axis that sustained the DSB (Niu *et al.* 2009; Subramanian *et al.* 2016). Activation of Mek1 kinase depends on the DSB-triggered phosphorylation of axis protein Hop1 by the ATM/ATR-related kinases Tel1/Mec1, which is thought to mediate Mek1 homodimerization and/or chromosomal recruitment (Schwacha and Kleckner 1997; Smith and Roeder 1997; Bailis and Roeder 1998; Niu *et al.* 2005; Carballo *et al.* 2008; Kim *et al.* 2010). Both Hop1 phosphorylation and its distribution along chromosome axes further mediate homolog bias, as suggested by the role in homolog bias of Hop1-chaperone Pch2 which carries out this function redundantly with Mec1^ATR^ (Joshi *et al.* 2015).

Intersister recombination is prevented at three additional stages: First, intersister exchange of early, low abundance DSBs is minimized by DNA helicase Mph1^FANCM^, which channels DSBs towards interhomolog repair by dissolving pre-pairing intersister D-loops (Sandhu *et al.* 2020). Second, cohesin Rec8 mediates homolog bias during SEI formation thereby promoting progression to interhomolog rather than intersister double Holliday junctions (Kim *et al.* 2010; Hong *et al.* 2013). Third, at the step of DSB second end capture, DNA helicase Sgs1^BLM^ prevents SEIs from re-invading a previously uninvolved chromatid (either the sister or the second homolog chromatid) (Oh *et al.* 2007).

### Processing of crossover-designated recombination intermediates

The predominant meiotic crossover pathway involves stable SEIs, DSB second end capture followed by repair synthesis of DNA previously removed during 5′ resection, dHJ formation and crossover-specific dHJ resolution (above; Fig. 4). Several steps along this pathway are carried out by meiotic paralogs of the mismatch repair machinery adapted to the meiotic process (Kunkel and Erie 2005). Following strand exchange, a heterodimer composed of the meiosis-specific MutS orthologs Msh4/5 (called MutSγ to distinguish it from the MutSα and MutSβ dimers involved in mismatch repair) stabilizes SEI intermediates and likely dHJs (Ross-Macdonald and Roeder 1994; Hollingsworth *et al.* 1995; Novak *et al.* 2001; Börner *et al.* 2004; Snowden *et al.* 2004; Jessop *et al.* 2006; Oh *et al.* 2007). MutSγ acts in collaboration with several meiosis-specific proteins collectively known as the “ZMM” group of proteins, which link recombination to assembly of the SC (Börner *et al.* 2004).

All ZMM proteins are cytologically associated with designated crossover sites where they mediate formation and/or stabilization of crossover-specific SEIs (Fig. 2, b and c) (Börner *et al.* 2004; Fung *et al.* 2004; Snowden *et al.* 2004). Besides MutSγ, the ZMMs include the Zip3 E3 SUMO ligase (Agarwal and Roeder 2000; Cheng *et al.* 2006; Serrentino *et al.* 2013), a sub-complex consisting of Zip2, Zip4, and Spo16 (ZZS) (Perry *et al.* 2005; Shinohara *et al.* 2008; De Muyt *et al.* 2018) as well as the Mer3 DNA helicase (Nakagawa and Ogawa 1999). Within the ZZS subcomplex, Zip2 and Spo16 are structurally related to the nucleotide excision repair endonuclease XPF-ERCC1 and, like MutSγ, bind branched DNA structures (Snowden *et al.* 2004; De Muyt *et al.* 2018). The presumed scaffolding factor Zip4 appears to provide a bridge between several ZMMs, such as Zip3, and axis protein Red1, as well as SC central element protein Ecm11 (De Muyt *et al.* 2018; Pyatnitskaya *et al.* 2022). Finally, the dually functioning transverse filament protein Zip1 mediates ZMM activity independent of its role as a structural component of the SC [for details see “*The functional relationship between SC and recombination*”].

While crossover-specific interhomolog SEIs are stabilized by ZMM proteins, these intermediates are dismantled by the DNA helicase Sgs1, which operates as a complex with Top3 and Rmi1 (STR complex) (Jessop *et al.* 2006; Jessop and Lichten 2008; De Muyt *et al.* 2012; Kaur *et al.* 2015; Tang *et al.* 2015). Competition between the ZMM and the STR complexes determines whether an interhomolog recombination intermediate is processed into a crossover or a non-crossover (Kaur *et al.* 2015; Tang *et al.* 2015).

ZMM-stabilized SEIs eventually are processed into interhomolog dHJs via the single-strand annealing activity of Rad52 which mediates capture of the second DSB end (Lao *et al.* 2008). For resolution of dHJs, MutSγ is joined at recombination sites by a MutL-related heterodimer Mlh1/3 [referred to as MutLγ, to distinguish it from the α and β mismatch repair dimers], which also binds dHJs and exhibits resolvase activity that exclusively gives rise to crossovers (Zakharyevich *et al.* 2012; Ranjha *et al.* 2014; Cannavo *et al.* 2020; Kulkarni *et al.* 2020; Sanchez *et al.* 2020). MutLγ is thought to resolve dHJs in a crossover-specific manner by nicking the DNA strands containing newly synthesized DNA in regions flanking the Holliday junctions (Fig. 4) (Kulkarni *et al.* 2020). Alternatively, a canonical Holliday junction resolution mechanism has been proposed that involves nicking of single-stranded regions at the two junction points (West *et al.* 2015; Cannavo *et al.* 2020).

Apart from MutSγ and MutLγ, dHJ resolution depends on several additional repurposed mismatch repair factors (Kunkel and Erie 2005). These include the sliding clamp PCNA (Pol30), which normally stabilizes DNA association of DNA polymerase, the heteropentameric PCNA-loader replication factor C (Rfc1-5), as well as exonuclease Exo1 (Kulkarni *et al.* 2020). The catalytic exonuclease activity of Exo1 is dispensable for stimulating MutLγ-mediated Holliday junction resolution (Zakharyevich *et al.* 2010, 2012; Kulkarni *et al.* 2020). Instead, Exo1 recruits the polo-like kinase Cdc5 to recombination sites, which activates dHJ resolution (Clyne *et al.* 2003; Sourirajan and Lichten 2008; Zakharyevich *et al.* 2012; Cannavo *et al.* 2020; Sanchez *et al.* 2020). The ZMM group of proteins may enforce a crossover outcome by mediating orientation-specific loading of PCNA during dHJ formation, thus directing the MutSγ/MutLγ/ExoI ensemble to nick specific DNA strands (Cannavo *et al.* 2020; Kulkarni *et al.* 2020).

### Non-crossover formation via synthesis-dependent strand annealing (SDSA)

The original model of DSB repair predicted that non-crossovers arise via nicking of the four crossing strands within the double Holliday junction (Szostak *et al.* 1983), yet several observations argue against this possibility for the bulk of meiotic non-crossovers. First, a majority of non-crossovers appear concurrently with, rather than after, dHJ formation, contradicting a precursor-product relationship (Allers and Lichten 2001a). Second, mutants with defects in the formation of SEIs and dHJs form non-crossovers normally, while crossovers are reduced or absent (Allers and Lichten 2001a; Börner *et al.* 2004). Third, crossovers and non-crossovers exhibit gene conversion tracts of different lengths, averaging 2 and 1.8 kb, respectively (Chen *et al.* 2008; Mancera *et al.* 2008; Ahuja *et al.* 2021), again suggesting that they are not derived from the same intermediate.

Non-crossovers are thought to arise by a process referred to as synthesis-dependent strand annealing (SDSA) involving interhomolog strand-exchange intermediates that are not stabilized by the ZMM complex (McMahill *et al.* 2007; De Muyt *et al.* 2012). During SDSA, these unstable D-loop intermediates undergo only limited repair synthesis with the homolog followed by displacement of the invading 3′ overhang (Fig. 4). Strand displacement is carried out by the combined action of the BLM-related DNA helicase Sgs1 in collaboration with decatenation complex Top3/Rmi1 (Jessop *et al.* 2006; Oh *et al.* 2007; Jessop and Lichten 2008; De Muyt *et al.* 2012; Zakharyevich *et al.* 2012; Kaur *et al.* 2015; Tang *et al.* 2015). Following its displacement, the 3′ extended single-stranded tail can anneal with the opposing DSB end giving rise to a non-crossover (Allers and Lichten 2001a; McMahill *et al.* 2007; Marsolier-Kergoat *et al.* 2018). Because only one of the two DSB ends engages in interhomolog strand exchange and repair synthesis, non-crossovers typically exhibit gene conversions towards one side of the DSB site (McMahill *et al.* 2007; Marsolier-Kergoat *et al.* 2018; Ahuja *et al.* 2021). Finally, a substantial fraction of non-crossovers (at least 25%) are generated via gap repair that fills in up to 200 bp between a pair of adjoining DSBs on the same DNA molecule (Johnson *et al.* 2021; Prieler *et al.* 2021).

### Processing of class II recombination events into crossovers and non-crossovers

**Fig. 5.**
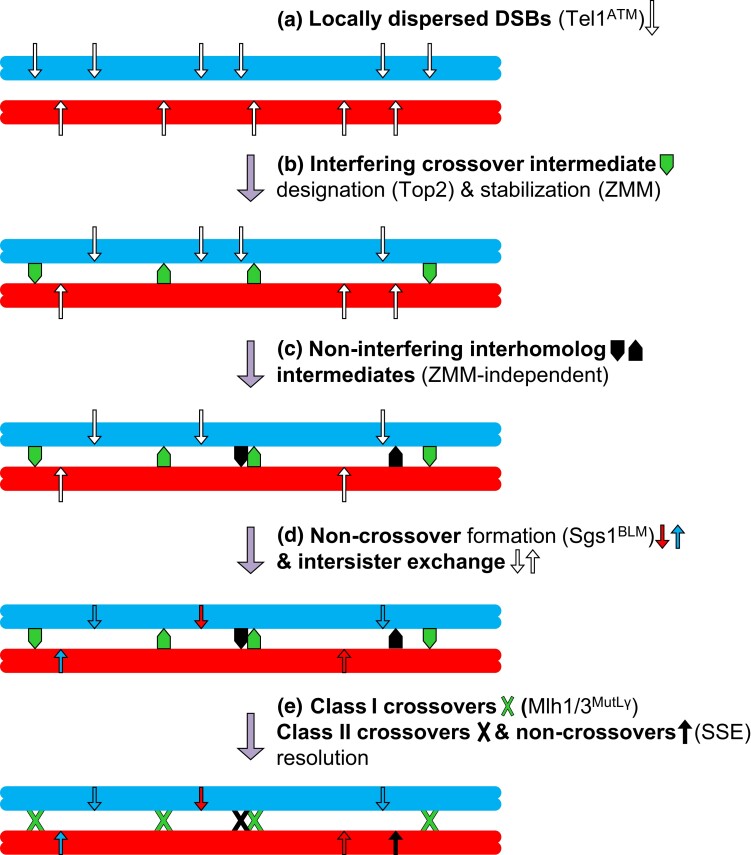
Quantitative contributions of distinct pathways to meiotic recombination. Diagram shows paired homologs in blue and red. Sister chromatids, shown here as a single unit, are equally likely to partake in the indicated recombination events. Steps in the diagram are functionally distinct but may occur contemporaneously or in a different order. Approximate contributions of four recombination outcomes are considered. Interhomolog and intersister recombination occurs at a ratio of 9:1. Interhomolog recombination events are divided between crossovers and non-crossovers at a ratio of 2:1. Class I and class II pathways contribute crossovers at a ratio of 2:1. a) ∼170 DSBs (long white arrows) are locally dispersed along the length of the chromosome through action of Tel1^ATM^ checkpoint kinase. b) A maximally spaced subset of early recombination intermediates, likely nascent strand exchange events, is designated as future interfering class I crossovers (green block arrows). Designation of class I crossover sites involves chromosome axis component Top2 while maintenance of their fate depends on the ZMM proteins. c) The interference-insensitive class II pathway contributes a subset of future crossovers (black block arrows), which involves structurally indistinguishable recombination intermediates as the class I crossover pathway. d) The remaining DSBs are processed into non-crossovers (short arrows filled with color of opposite homolog) or into intersister exchanges (short open arrows). e) Resolution of class I crossovers via MutLγ (green X) and of class II recombination events (black X) by structure-selective resolvases including Mms4-Mus81. Some class II events also give rise to non-crossovers (not shown).

A small fraction of interhomolog dHJs may form independently of ZMM proteins in wild-type cells, and resolve via an alternative, so-called class II pathway; this pathway dominates in absence of Sgs1 or ZMM group proteins (above; Fig. 5) (De Muyt *et al.* 2012). The class I and class II recombination pathways are thought to deviate during or after the DSB first end strand exchange (Börner *et al.* 2004; De Muyt *et al.* 2012). D-loop intermediates not stabilized as SEIs by ZMM proteins are normally subject to dissociation by Sgs1. When dissociation fails, ZMM-independent SEIs progress to dHJs that are subsequently resolved by structure selective endonucleases (SSE; Mms4/Mus81, Slx1/4 as well as Yen1) and independently of the MutLγ complex (de los Santos *et al.* 2003; Börner *et al.* 2004; Oh *et al.* 2008; De Muyt *et al.* 2012; Zakharyevich *et al.* 2012). Resolution of class II crossovers further depends on Smc5/6, a repair-specific SMC complex related to cohesin and condensin (Copsey *et al.* 2013; Lilienthal *et al.* 2013; Xaver *et al.* 2013).

Unlike ZMM-associated “class I” dHJ intermediates, which predominantly generate crossovers, dHJs formed by the class II pathway are resolved in an unbiased manner by SSEs, equally giving rise to crossovers and non-crossovers (De Los Santos *et al.* 2003; Börner *et al.* 2004; Oh *et al.* 2008; De Muyt *et al.* 2012; Zakharyevich *et al.* 2012). SSE normally become active only following exit from prophase I through phosphorylation by Cdc5 and CDK (Matos *et al.* 2011). Yen1 in particular is subject to inhibitory phosphorylation until meiosis II and, being dispensable during wild-type meiosis, is thought to serve as a resolvase of last resort (Matos *et al.* 2011). Whereas “class I” crossovers exhibit a patterned genome-wide distribution, as indicated by their maximum spacing (interference) and assurance that chromosomes independent of size undergo at least one crossover, crossovers formed along the alternative “class II” pathway lack both of these features (for details see “*Spatial and temporal control of recombination*”) (Sym and Roeder 1994; Novak *et al.* 2001; de los Santos *et al.* 2003).

## Homolog pairing and reinforcement of chromosome alignment

### Role of recombination in homolog pairing

Alignment of homologous chromosomes in pairs occurs in close coordination with recombination progression. Although homologs associate at some levels in vegetative nuclei, they achieve exclusive and intimate alignment only during mid-meiotic prophase I (Scherthan *et al.* 1994; Weiner and Kleckner 1994; Nag *et al.* 1995; Burgess *et al.* 1999; Peoples *et al.* 2002; Sandhu *et al.* 2020). Both genetic and cytological approaches indicate that stable homolog pairing strongly depends on early steps in meiotic recombination, i.e. DSB formation and strand exchange (Loidl *et al.* 1994; Weiner and Kleckner 1994; Peoples *et al.* 2002; Peoples-Holst and Burgess 2005; Lui *et al.* 2006). Yet, homolog recognition does not appear to be solely underpinned by strand exchange, as mutants lacking both Rad51 and Dmc1 exhibit a substantial level of homolog pairing compared to the low level observed in a *spo11* mutant (Tsubouchi and Roeder 2003; Yisehak and Macqueen 2018).

Strand exchange only establishes homologous interactions at a local sequence level, suggesting that additional layers of regulation exist to minimize pairing between homologous regions on heterologous chromosomes. While recombination between such regions occurs at substantial frequencies, these interactions normally do not impede the stable alignment of homologs (Jinks-Robertson and Petes 1985; Lichten *et al.* 1987; Haber *et al.* 1991; Goldman and Lichten 1996, 2000; Jinks-Robertson *et al.* 1997). Interestingly, in the absence of Dmc1-accessory protein dimer Hop2–Mnd1, some pairing and SC assembly occur between heterologous chromosomes (Leu *et al.* 1998; Peoples *et al.* 2002; Tsubouchi and Roeder 2002), and this erroneous pairing is mediated by Rad51 or Dmc1 recombinases, at least in certain strain backgrounds (Tsubouchi and Roeder 2003; Zierhut *et al.* 2004). Hop2 and Mnd1 thus appear to facilitate recombinase discrimination between homologous and heterologous chromosomes.

### Recombination-independent pairing mechanisms

Several recombination-independent processes modulate chromosome associations during prophase I. These include homology-independent centromere coupling, formation of a “bouquet” organization, and actin-mediated rapid chromosome movements. Such meiosis-specific chromosome redistribution mechanisms may improve the capacity of recombination pathways to align partner chromosomes.

#### Leptotene centromere coupling

Centromeric regions of leptotene chromosomes associate in pairwise fashion, independent of homology or Spo11 but dependent on Rec8-cohesin and the SC transverse filament protein Zip1 (Tsubouchi and Roeder 2005; Falk *et al.* 2010; Obeso and Dawson 2010). The function of this so-called centromere coupling is not understood, but centromere associations may be non-random, as chromosome conformation capture experiments suggest they are guided by chromosome size, and thus may presort chromosomes for homolog pairing (Lefrancois *et al.* 2016). Release from homology-independent centromere coupling depends on recombination initiation, N-terminal Zip1 phosphorylation mediated by Mec1^ATR^, and a fully functional proteasome (Tsubouchi and Roeder 2005; Falk *et al.* 2010; Obeso and Dawson 2010; Ahuja *et al.* 2017).

#### Telomere bouquet

In non-meiotic interphase cells, centromeres are clustered as a remnant of the preceding cell division in the so-called “Rabl” configuration (Scherthan *et al.* 1994; Zickler and Kleckner 1998). The meiotic bouquet refers to a transient reorganization within the zygotene nucleus where telomeres cluster at a limited region of the nuclear envelope near the spindle pole body (Jin *et al.* 1998; Trelles-Sticken *et al.* 1999). Bouquet formation requires a telomere-associated protein complex containing Ndj1, Csm4, and the SUN-domain protein Mps3, which form a bridge through the nuclear envelope that connects the ends of meiotic chromosomes with cytoplasmic actin cables (Conrad *et al.* 1997, 2007, 2008; Trelles-Sticken *et al.* 2000, 2005; Kosaka *et al.* 2008; Wanat *et al.* 2008).

#### Rapid prophase movements (RPMs)

The bouquet-promoting proteins Ndj1, Csm4, and Mps3 also facilitate rapid, actin-mediated chromosome movements starting early in meiotic prophase with average speeds of ∼0.4 μm per second in a nucleus measuring ∼3 μm in diameter (Trelles-Sticken *et al.* 2005; Scherthan *et al.* 2007; Conrad *et al.* 2008; Koszul *et al.* 2008). *ndj1*, *csm4*, and *mps3* mutants exhibit delays in homolog pairing and recombination (Conrad *et al.* 1997; Trelles-Sticken *et al.* 2000; Wu and Burgess 2006; Kosaka *et al.* 2008; Koszul *et al.* 2008; Wanat *et al.* 2008; Rao *et al.* 2011; Lee *et al.* 2012) and, in strains expressing non-null *mps3* alleles, pairing outcomes correlate with RPMs but not bouquet formation (Lee *et al.* 2012). RPMs may actively promote chromosomal encounters, as encounter frequencies between both homologous and non-homologous chromosomes are substantially reduced in *csm4* mutants (Lee *et al.* 2012). Alternatively, or in addition, RPMs could serve a homology stringency test function by pulling apart non-allelic interactions (Conrad *et al.* 2008; Koszul *et al.* 2008; Koszul and Kleckner 2009). That RPMs reach maximal speed in pachytene-arrested *ndt80* mutant cells is consistent with a role in dismantling non-allelic interactions (Conrad *et al.* 2008; Kosaka *et al.* 2008; Koszul *et al.* 2008; Wanat *et al.* 2008). Finally, recombination also affects chromosome movements, either directly or indirectly, as RPMs do not reach wild-type speed in the absence of recombination (Conrad *et al.* 2008; Kosaka *et al.* 2008; Koszul *et al.* 2008; Wanat *et al.* 2008).

### The SC: an outcome of successful homolog pairing

Meiotic chromosome structure is modified during the zygotene-to-pachytene transition through the process of synapsis, which entails the assembly of a macromolecular protein structure called the synaptonemal complex (SC) (Fig. 2, b and c). While the SC is dispensable for homolog pairing, it promotes an intimate physical association between partner chromosome axes along their entire length (Weiner and Kleckner 1994; Rockmill, Sym, *et al.* 1995). The SC forms a ∼100 nm wide bridge between chromosome axes through the multimerization of SC central region proteins, which include rod-like transverse filament and central element proteins (Sym *et al.* 1993; Zickler and Kleckner 1999; Humphryes *et al.* 2013; Voelkel-Meiman *et al.* 2013).

Transverse filaments in yeast are composed of the Zip1 protein, which carries an extensive central coiled-coil region that allows the formation of dimers or tetramers. Parallel dimer or tetramer units of Zip1 arrange in mirror-image fashion between aligned homologous axes, with their N termini overlapping at the SC midline (Dong and Roeder 2000; Voelkel-Meiman *et al.* 2013). The length of Zip1 coiled-coil units determines the width of the SC (Sym and Roeder 1995; Tung and Roeder 1998; Dong and Roeder 2000). A central element protein complex, composed of Ecm11 and Gmc2, organizes transverse filaments at the midline of the SC, a function dependent on Ecm11 SUMOylation (Humphryes *et al.* 2013; Voelkel-Meiman *et al.* 2013). Although cytological images give the impression of a fixed zipper-like structure, the SC is dynamic in nature as suggested by the capacity of the central region to dissolve and reassemble upon transient exposure to aliphatic alcohols (Rog *et al.* 2017). Furthermore, SC central region building block proteins continuously accumulate between homolog axes during the pachytene stage, again indicating that the SC is not a static structure (Voelkel-Meiman *et al.* 2012, 2016).

Although SC-like structures can form between nonhomologous chromosomes or even between sister chromatids (Loidl *et al.* 1991; Leu *et al.* 1998; Voelkel-Meiman *et al.* 2012), SC normally assembles between homologs, downstream of recombination initiation and homolog pairing (Sym *et al.* 1993). Thus, the extent of SC assembly depends strongly on early recombination events and fails altogether in *spo11* mutants (Giroux *et al.* 1989; Henderson and Keeney 2004; Macqueen and Roeder 2009). In recombination-deficient cells, SC proteins instead self-assemble near the nucleolus into a singular aggregate called the polycomplex, which frequently appears to retain the tripartite structure of the SC (Klapholz *et al.* 1985; Sym and Roeder 1995). SC assembly is not restored to *spo11* meiotic cells supplied with multiple DSBs generated by the HO endonuclease or arising from phleomycin exposure (Yisehak and Macqueen 2018), raising the possibility that Spo11-initiated recombination uniquely interfaces with the synapsis machinery.

Although most SC assembly events initiate at recombination sites (Fung *et al.* 2004; Henderson and Keeney 2004), the earliest SC assembly initiates at recombination-suppressed centromere regions (Tsubouchi *et al.* 2008). Synapsis initiation at centromeres is mechanistically distinct from the one operating at recombination sites. For example, while required for synapsis from recombination sites, the E3 ligase Zip3 is dispensable for SC assembly at centromeres. Conversely, in *spo11* mutants, Zip3 together with the Fpr3 prolyl isomerase prevents unregulated SC assembly from centromeres (Macqueen and Roeder 2009).

### ZMM proteins link recombination to SC assembly

ZMM proteins not only promote crossover recombination but also couple recombination physically and mechanistically to SC assembly (Agarwal and Roeder 2000; Börner *et al.* 2004; Tsubouchi *et al.* 2006; Shinohara *et al.* 2008). The E3 ligase Zip3, the endonuclease XPF-ERCC1-related ZZS subcomplex (Zip2, Zip4, Spo16), and the MutSγ complex co-localize with one another and with SC central region proteins (Zip1, Ecm11, Gmc2) at recombination sites, constituting the synapsis initiation complex (Fung *et al.* 2004). Several components of the ZMM group link the recombination complex with the SC and/or chromosome axis. Zip4 creates a physical link between recombination, the axis and the SC central region, as it interacts with both axis protein Red1 and SC central element protein Ecm11 (Humphryes *et al.* 2013; De Muyt *et al.* 2018; Arora and Corbett 2019; Pyatnitskaya *et al.* 2022). In addition, Zip3 mediates SUMOylation of axis protein Red1, which contributes to timely synapsis (Cheng *et al.* 2006; Eichinger and Jentsch 2010). Zip1 and Zip3 also mediate phosphorylation of Msh4 at its N-terminal degron region by DDK, protecting Msh4 from proteasomal degradation, and both Zip1 and Zip3 promote SUMOylation of Msh4 (He *et al.* 2020, 2021).

While ZMM complex proteins colocalize, they appear to have somewhat different effects on SC assembly. SC formation is abolished in mutants missing the ZZS complex (Chua and Roeder 1998; Tsubouchi *et al.* 2006; Shinohara *et al.* 2008), yet synapsis is only diminished and delayed in MutSγ mutants and in *zip3* mutants, with some effects of strain background and incubation conditions (Agarwal and Roeder 2000; Börner *et al.* 2004). The relatively mild synapsis defect of *zip3* mutants can largely be explained by Zip3's opposing roles in synapsis regulation at different chromosomal sites: whereas Zip3 promotes SC assembly from recombination sites, it prevents SC assembly from centromeres (Tsubouchi *et al.* 2008; Macqueen and Roeder 2009; Voelkel-Meiman *et al.* 2012). Finally, mutations in several additional factors, including the proteasome and protein phosphatase 4, share with *zmm* mutants defects in recombination, synapsis, and meiotic progression (Falk *et al.* 2010; Ahuja *et al.* 2017). These factors may act on ZMM proteins or contribute to additional pathways that coordinately affect recombination and synapsis.

### The functional relationship between SC and recombination

Although SC central region proteins from different species exhibit limited sequence similarity, they invariably align homolog axes at a distance of ∼100 nm (Page and Hawley 2004). The SC's conserved width may be related to the tight functional relationship between SC proteins and recombination. In light of this possibility, it is intriguing that the average inter-junction distance of double Holliday junctions corresponds to ∼90 nm of B-form DNA (Cromie *et al.* 2006; Oh *et al.* 2008).

When Zip1 from the closely related yeast *Kluyveromyces lactis* is expressed in place of Zip1 from *S. cerevisiae*, it fails to support SC assembly, but still mediates double Holliday junction stabilization and crossover formation (Voelkel-Meiman *et al.* 2015). Furthermore, the absence of SC structural proteins Ecm11 and Gmc2 leads to excess MutSγ-mediated crossovers (Voelkel-Meiman *et al.* 2016). Thus, the SC structure is dispensable for meiotic recombination in budding yeast, and instead is required for limiting crossover recombination (Voelkel-Meiman *et al.* 2013, 2016). The anti-recombination function of budding yeast SC is explained at least in part by the capacity of SC central region proteins (i.e. Zip1 and Ecm11–Gmc2) to prevent excess DSBs (Thacker *et al.* 2014; Subramanian *et al.* 2016; Mu *et al.* 2020; Lee *et al*. 2021).

Although SC is dispensable for crossover recombination, it nevertheless forms the physical context for crossover-fated recombination intermediates, as evidenced by the localization of ZMM as well as MutLγ foci to the midline of SC structures (Agarwal and Roeder 2000; Novak *et al.* 2001; Voelkel-Meiman *et al.* 2019; Sanchez *et al.* 2020). While the function of the SC is presently unknown, it may serve a chaperone-like role in regulating interactions between proteins and/or DNA structures at recombination sites. Accordingly, SC central region proteins regulate aspects of recombination intermediate processing such as dHJ resolution, gene conversion tract length and continuity, as well as robust mismatch repair (Rockmill *et al.* 2013; Oke *et al.* 2014; Lee *et al.* 2021; Voelkel-Meiman *et al.* 2022).

Intriguingly, the SC transverse filament protein Zip1 serves a genetically-separable role in promoting MutSγ-mediated crossovers (Voelkel-Meiman *et al.* 2016; Voelkel-Meiman *et al.* 2019) and thus is itself classified as a ZMM factor (Börner *et al.* 2004). Adjacent regions within Zip1's N terminus independently promote either recombination or SC assembly, identifying this Zip1 domain as a regulatory hub that couples recombination and synapsis (Voelkel-Meiman *et al.* 2019). A role of Zip1 in linking recombination and synapsis is further suggested by coordinate effects on both processes of Cdc7-mediated, C-terminal Zip1 phosphorylation (Chen *et al.* 2015).

## Spatial and temporal control of recombination

### Recombination frequencies vary between genome regions

Around 90 crossovers are detected per meiotic nucleus in marker-rich hybrid strains, corresponding to a frequency of ∼7 crossovers per megabase (or ∼350 cM/Mb, compared to an average genetic map distance of 1 cM/Mb in humans) (Chen *et al.* 2008; Mancera *et al.* 2008). The ∼65 non-crossovers per meiotic nucleus occur in the same regions as crossovers, although there are regions with considerable biases towards either the crossover or the non-crossover outcome (Chen *et al.* 2008; Mancera *et al.* 2008). Finally, of the ∼170 DSBs in a given yeast nucleus, an estimated 15 undergo repair with the sister chromatid, although these recombination events are difficult to quantify as they do not leave traces in gamete genomes (Fig. 5) (Chen *et al.* 2008; Mancera *et al.* 2008; Marsolier-Kergoat *et al.* 2018).

Crossover rates vary more than 100-fold along the yeast genome, defining “hot” and “cold” regions. Frequencies of interhomolog recombination events largely correlate with DSB frequencies (Marsolier-Kergoat *et al.* 2018). While DSB hotspots tend to be associated with open chromatin, divergent promoters, GC content, and specific histone modifications, the factors that make some hotspots hotter than others remain poorly understood (Blitzblau *et al.* 2007; Buhler *et al.* 2007; Pan *et al.* 2011; Zhu and Keeney 2015; Gothwal *et al.* 2016). At a regional scale, DSB levels are inversely correlated with axial element-associated proteins including Red1/Hop1 as well as RMM (Blat *et al.* 2002; Panizza *et al.* 2011; Sun *et al.* 2015). Accordingly, the larger chromosome context may influence DSB frequency of a given hotspot sequence (Borde *et al.* 1999).

The three shortest yeast chromosomes exhibit notably higher DSB and crossover frequencies than the rest of the genome (Kaback *et al.* 1992; Blitzblau *et al.* 2007; Pan *et al.* 2011), a feature correlated with longer lasting recruitment of DSB formation factors such as Rec114 (Murakami *et al.* 2020). Increased DSB frequencies along shorter chromosomes are determined by intrinsic sequence elements as inferred from unchanged DSB frequencies when a smaller chromosome is fused to a larger one (Mu *et al.* 2020; Murakami *et al.* 2020). In addition, DSB formation in large (∼100 kb) chromosome-end adjacent regions is enhanced via increased retention of axis protein Hop1 in these regions (Subramanian *et al.* 2019).

In subtelomeric and pericentromeric regions as well as within the rDNA repeat cluster on yeast chromosome XII and adjacent regions, DSB and/or crossover frequencies are below average (San-Segundo and Roeder 1999; Chen *et al.* 2008; Mancera *et al.* 2008; Pan *et al.* 2011; Vader *et al.* 2011; Subramanian *et al.* 2019). DSBs in pericentromeric regions are kept at low levels by kinetochore proteins (Vincenten *et al.* 2015). Pericentromeric crossovers are further suppressed by Rec8- and Zip1-mediated direction of DSB repair towards the sister chromatid instead of the homolog (Lambie and Roeder 1988; Chen *et al.* 2008). In the genome region containing the rDNA repeat cluster, DSB formation is repressed through the histone deacetylase Sir2 and the condensin complex (Gottlieb and Esposito 1989; San-Segundo and Roeder 1999; Li *et al.* 2014). Sir2 likely acts by excluding the axis protein Hop1 from the rDNA (Gottlieb and Esposito 1989; San-Segundo and Roeder 1999). Intriguingly, in regions adjacent to the rDNA cluster, Sir2 has a DSB-inducing effect that is counteracted by the AAA-ATPase Pch2 and the origin-recognition complex factor Orc1, which together help remove Hop1 from chromosome axes in these regions (Vader *et al.* 2011; De Ioannes *et al.* 2019).

### Control of crossover distribution

Each homolog pair, independent of size, must acquire at least one crossover to ensure homolog disjunction toward opposite spindle poles during meiosis I. If crossover placement followed a Poisson distribution, smaller chromosomes would frequently fail to acquire a crossover resulting in homolog missegregation (Kaback *et al.* 1992; Sym and Roeder 1994). The molecular pathway(s) that ensure formation of at least one (obligatory) chiasma per homolog pair are referred to as “crossover assurance” (Pazhayam *et al.* 2021). They likely include enhancements of DSB formation along small chromosomes and of interhomolog bias (above). At least two additional mechanisms dictate the genome-wide distribution pattern of crossovers. First, crossover interference, originally discovered when creating the first chromosome linkage maps in *Drosophila* (Sturtevant 1913), is a phenomenon whereby a given crossover reduces the likelihood of additional crossovers in nearby intervals, resulting in regular crossover spacing along homolog pairs (Pazhayam *et al.* 2021). Second, crossover homeostasis preferentially generates crossovers at the expense of non-crossovers when DSBs are limiting and/orhomolog bias is weak (Martini *et al.* 2006; Lao *et al.* 2013; Sandhu *et al.* 2020). The same mechanism appears to maintain the number of synapsis initiation sites at high levels (Henderson and Keeney 2004).

#### Crossover interference and crossover assurance

A first level of maximum spacing between recombination sites is established by mechanisms that prevent the clustered formation of DSBs along the same chromatid (Garcia *et al.* 2015). This DSB interference depends on activity of the ATM-like kinase Tel1 and extends over chromosome regions of at least 70 kb, but no more than 150 kb (Garcia *et al.* 2015). At a later step, though no later than DSB strand exchange, the interfering distribution of crossover-fated intermediates along chromosomes is established via a pathway that targets the catalytic activity of type-II topoisomerase Top2 via the SUMO and/or ubiquitin system (Zhang *et al.* 2014). Interference patterning of crossovers thus is controlled by proteins that constitute the meiotic chromosome axis, including Top2, which prominently localizes along the length of meiotic chromosomes and promotes a structural transition of chromosome axes (Klein *et al.* 1992; Borner *et al*. 2004; Heldrich *et al.* 2020). This pathway requires Sir2, though not its histone deacetylase activity, which recruits the heterodimeric SUMO-targeted ubiquitin ligase Slx5/8. It also requires SUMOylation of Top2 and interaction with SUMO of the axis protein Red1 (Zhang *et al.* 2014).

Both cytological and genetic measurements suggest that interference in budding yeast extends across ∼130 kb (corresponding to ∼0.4 micron of pachytene chromosome length) (Zhang *et al.* 2014). One proposed mechanism for crossover interference involves the establishment of physical tension along the semi-elastic chromosome axis, which is alleviated by a discontinuity in the axis—the site of the flaw being the site of crossover commitment, followed by maturation into an actual crossover. According to this model, relief of tension prevents additional crossovers over a certain distance (Kleckner 2006).

Until crossover-specific resolution of Holliday junctions has been completed, the crossover fate of interference-distributed strand exchange intermediates needs to be maintained, a task performed by ZMM proteins Zip1, Msh4/5, and Mer3, but independent of ZZS subcomplex components Spo16 and Zip4 (above) (Borner *et al*. 2004; Shinohara *et al.* 2008). Zip3 focus distribution indicates that crossover interference is correctly established in *zip1* and other *zmm* mutants, even though crossovers detected in the resulting gametes do not exhibit an interference distribution (Sym and Roeder 1994; Nakagawa and Ogawa 1999; Novak *et al.* 2001; Fung *et al.* 2004; Zhang *et al.* 2014). Accordingly, ZMMs are dispensable for the establishment of interference, but critical for ensuring that crossover-designated ("class I") intermediates are successfully processed into crossovers (Fig. 5; Börner *et al.* 2004).

Non-interfering crossovers formed in absence of ZMM proteins are referred to as class II crossovers, to distinguish them from class I crossovers that exhibit interference (De Los Santos *et al.* 2003; Börner *et al.* 2004). Consistent with the idea that the class II recombination pathway is also active during wild-type meiosis, ∼70 class I ZMM foci per nucleus are observable cytologically, but ∼90 crossovers are detected genetically (Fung *et al.* 2004; Chen *et al.* 2008; Mancera *et al.* 2008; Joshi *et al.* 2009). Thus, the class II recombination pathway likely contributes about one fifth of crossovers, as further indicated by detection of ∼15 foci of the class II pathway resolvase Mms4 (Copsey *et al.* 2013). While dHJs formed along the class I pathway are resolved by MutLγ and its interaction partners, dHJs formed along the class II pathway are resolved by SSE Mms4/Mus81 as well as Slx1/4 (see “*Processing of class II recombination events into crossovers and non-crossovers*”) (De Muyt *et al.* 2012; Zakharyevich *et al.* 2012). Consistent with activity of Mms4/Mus81 along the class II pathway, crossover interference remains intact in the *mms4* mutant, even though crossovers are substantially decreased, with additive effects of *msh5* on crossover reduction (De Los Santos *et al.* 2003; Argueso *et al.* 2004). Consistent with a proposed role for the Sgs1 helicase in channeling recombination intermediates away from the class II and into the ZMM-dependent class I recombination pathway, crossover interference is impaired in the *sgs1* mutant (Oh *et al.* 2007) and absence of Sgs1 results in increased crossover formation in *zip1Δ* (Jessop *et al.* 2006). Impaired interference in mutants that lack Dmc1 or the Dmc1-activator Tid1 further highlights the importance of appropriate DSB strand exchange for crossover interference (Shinohara *et al.* 2003; Lao *et al.* 2013).

#### Crossover homeostasis

At low DSB abundance, for example during early meiosis, or when DSBs are reduced in a hypomorphic *spo11* mutant, a higher proportion of DSBs is repaired with the sister chromatid, likely because the homolog remains inaccessible due to incomplete recombination-dependent homolog pairing (Joshi *et al.* 2015; Sandhu *et al.* 2020). Among the remaining interhomolog recombination events, crossovers are enhanced at the expense of non-crossovers, a process referred to as crossover homeostasis (Martini *et al.* 2006; Sandhu *et al.* 2020). At the same time, high DSB levels are insufficient to ensure the obligate crossover, as certain *zmm* mutants frequently exhibit non-exchange chromosomes (E0 events) despite elevated DSB levels, likely because the class II recombination pathway lacks most aspects of crossover control, including crossover interference, assurance and homeostasis (Chen *et al.* 2008).

### Downregulation of interhomolog recombination in late prophase I

At late prophase I, recombination is gradually attenuated through downregulation of DSB formation, and shifts from strong homolog bias again back to increased intersister repair (Thacker *et al.* 2014; Subramanian *et al.* 2016). This process is chromosome-autonomous and is strongly linked to Zip1 protein function. The effect of Zip1 is mediated in part through the recruitment of Pch2 and subsequent Pch2-dependent removal of Hop1, which results in a drop in DSB activity and releases the inhibition of intersister strand exchange by protein kinase Mek1 (San-Segundo and Roeder 1999; Börner *et al.* 2008; Subramanian *et al.* 2016).

### The recombination checkpoint

Throughout prophase I, cells monitor the presence of stalled recombination complexes and/or unsynapsed chromosome axes using a surveillance mechanism that is often referred to as the recombination checkpoint or pachytene checkpoint (Xu *et al.* 1997; Macqueen and Hochwagen 2011; Subramanian and Hochwagen 2014; Raina *et al.* 2023). Prophase I delay/arrest triggered by this checkpoint depends on signaling by the DNA damage sensor kinases Tel1 and Mec1, and thus shares fundamental features with the canonical DDR network (Lydall *et al.* 1996; Usui *et al.* 2001). Tel1 responds primarily to Spo11-linked DNA ends whereas Mec1 relies on accessory factors, including Ddc2 and the Rad17/Mec3/Ddc1 complex, to sense ssDNA and ssDNA/dsDNA junctions, respectively (Usui *et al.* 2001; Hong and Roeder 2002; Refolio *et al.* 2011). Importantly, because unsynapsed regions continue to form DSBs, this checkpoint is also activated by defects in chromosome synapsis (San-Segundo and Roeder 1999; Hong and Roeder 2002; Thacker *et al.* 2014). Paradoxically, the absence of recombination intermediates (e.g. in *spo11*) does not trigger meiotic arrest, and indeed bypasses arrest in recombination defective mutants (McKee and Kleckner 1997a). Compared to the DDR, the substrate spectrum of Mec1 and Tel1 in the recombination checkpoint is greatly expanded to include numerous meiosis-specific proteins, presumably to help coordinate the progression of DSB repair with other meiotic processes (Kar *et al.* 2022). Recombination surveillance likely involves meiotic axis proteins, which, by a poorly understood mechanism dampen the activation of the canonical DDR effector kinase Rad53 and instead help activate the meiotic Rad53 paralogue Mek1, via the phosphorylation of HORMA domain protein Hop1, with possible involvement of a cytoplasmic component (Lydall *et al.* 1996; Carballo *et al.* 2008; Cartagena-Lirola *et al.* 2008; Herruzo *et al.* 2021). In addition to their monitoring function, checkpoint components also modulate the underlying recombination reaction, indicating that arrest bypass in checkpoint mutants may also involves changes in the monitored process (Grushcow *et al.* 1999; Börner *et al.* 2008). The recombination checkpoint is notably less sensitive to DSBs than the canonical DDR: Indeed, a single DSB fails to trigger a detectable response during meiotic prophase, and even dozens of persistent breaks in some cases fail to cause a terminal arrest (Malkova *et al.* 1996; Hochwagen *et al.* 2005).

## Exit from prophase I

At the mid to late pachytene stage, activation of the meiosis-specific transcription factor Ndt80 leads to the increased expression of ∼150 “middle” genes. These middle genes encode factors that promote cellular events required for prophase exit, chromosome segregation, and spore morphogenesis (Xu *et al.* 1995; Chu *et al.* 1998; Primig *et al.* 2000).

### Hallmark events of prophase I exit are triggered by polo-like kinase Cdc5

**Fig. 6.**
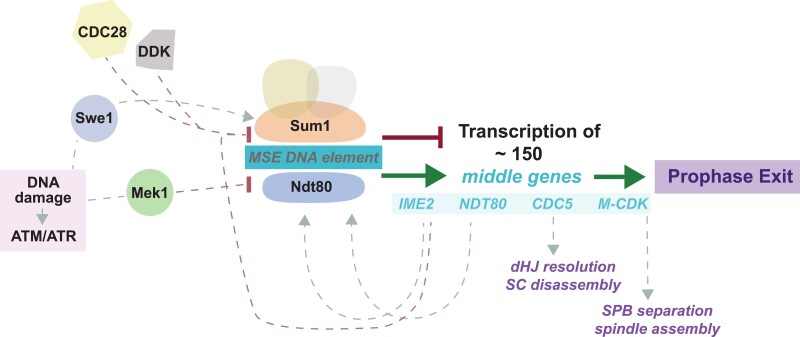
Signaling pathways at the exit from prophase I. Multiple signals control prophase exit by altering the capacity of Sum1 and Ndt80 to bind the Middle Sporulation DNA sequence Element (MSE). Illustration depicts positive and negative signals that control Ndt80 transcription factor activity. The Sum1 transcriptional repressor complex competes with Ndt80 for binding the MSE DNA element in the promoter regions of ∼150 genes. As meiotic prophase progresses, Ime2, Cdc28, and DDK kinase activities render Sum1 less capable of binding the MSE, while Ime2 activity stimulates Ndt80 binding the MSE. Proteins encoded by Ndt80 target genes (the “middle genes”) include Ime2 and Ndt80, both of which bolster the prophase exit circuit, the Cdc5 polo-like kinase, which promotes dHJ resolution and SC disassembly, and M-Cdk kinase, which promotes spindle pole body (SPB) separation. The prophase exit pathway is modulated by checkpoint signals: Unrepaired meiotic DSBs activate Mec1^ATR^/Tel1^ATM^ kinases, which in turn activate the Swe1 and Mek1 kinases. Swe1 stimulates Sum1 repressor activity, while Mek1 inhibits Ndt80 activity.

Exit from prophase I comprises an eventful cell-cycle transition involving: i) disassembly of SC and axial element structures, ii) resolution of double-Holliday-junctions, and iii) spindle-pole-body separation in preparation for formation of the meiosis I spindle (Fig. 6) (Shuster and Byers 1989; Xu *et al.* 1995). Induction of polo-like kinase Cdc5 by Ndt80 is sufficient to trigger several of these key prophase I exit events (Clyne *et al.* 2003; Sourirajan and Lichten 2008). Cdc5 likely stimulates SC disassembly at least in part by destabilizing the axial-element component Red1 (Clyne *et al.* 2003; Sourirajan and Lichten 2008; Prugar *et al.* 2017; Sanchez *et al.* 2020). Cdc5 further promotes Holliday junction resolution by associating with and activating MutLγ (Sanchez *et al.* 2020) as well as Mus81–Mms4 resolvases (De Los Santos *et al.* 2003; Jessop and Lichten 2008; Matos *et al.* 2011). Furthermore, Cdc5-dependent hyper-phosphorylation inhibits the Sgs1 DNA helicase, which potentially shifts recombination outcomes (Grigaitis *et al.* 2020). On the other hand, Cdc5 is not sufficient to promote spindle pole body separation during prophase exit. This event is instead promoted by M-phase CDK (Sourirajan and Lichten 2008), whose regulatory B-type cyclin components (Clb1, Clb4) are encoded by Ndt80 target genes (Chu and Herskowitz 1998; Leu and Roeder 1999).

### Control of Ndt80-mediated middle-gene expression

Middle-gene (i.e. post-pachytene) expression is facilitated by an increase in Ndt80 activity (itself encoded by a middle gene) (Chu and Herskowitz 1998; Tung *et al.* 2000; Pak and Segall 2002) and the downregulation of the Sum1/Rfm1/Hst1 transcriptional repressor complex (Fig. 6) (Xie *et al.* 1999; Lindgren *et al.* 2000; Mccord *et al.* 2003). The Sum1/Rfm1/Hst1 repressor competes with Ndt80 for binding a DNA sequence element called the middle-sporulation element (MSE) in the promoters of middle genes (Winter 2012). Sum1 directly binds the MSE and is regulated by multiple kinases including Ime2, whose activity increases toward mid-prophase (Primig *et al.* 2000; Benjamin *et al.* 2003; Ahmed *et al.* 2009). Cdc28 also targets Sum1, which primes Sum1's further phosphorylation by DDK (Sopko *et al.* 2002; Lo *et al.* 2008; Corbi *et al.* 2014). Accumulation of phosphates eventually renders the Sum1 complex unable to bind the MSE, resulting in an increase in *NDT80* transcripts (Pierce *et al.* 2003; Corbi *et al.* 2014). Conversely, phosphorylation by Ime2 renders the Ndt80 protein more effective at binding DNA and stimulating gene expression (Sopko *et al.* 2002; Benjamin *et al.* 2003; Sopko and Stuart 2004). Finally, Ndt80 increases transcription of *IME2* and *CDC5*, which further stimulates Ndt80 activity in a feed-forward loop (Fig. 6) (Benjamin *et al.* 2003; Acosta *et al.* 2011; Gonzalez-Arranz *et al.* 2021).

### Control of prophase I exit

Several mechanisms collaborate to ensure that cells do not exit prematurely from prophase I. Ama1, a meiosis-specific activator of the anaphase-promoting complex/cyclosome (APC/C), targets key proteins for proteasomal degradation that may otherwise destabilize prophase chromosomal structures and promote entry into metaphase I, including the cell-cycle regulators Ndd1 and Cdc5 (Okaz *et al.* 2012). Ama1 thereby renders meiotic cells dependent on Ndt80 for progression beyond the pachytene stage and through prophase exit. Ndt80 activity is in turn attenuated by the recombination checkpoint, which prevents or delays exit from prophase I in response to unprocessed recombination intermediates (above). To activate this checkpoint, the canonical DNA-damage sensor kinases Mec1^ATR^ and Tel1^ATM^ activate Mek1 kinase by phosphorylating its binding partner Hop1 early during meiosis, with additional effects on recombination partner choice (above) (Niu *et al.* 2005, 2007; Carballo *et al.* 2008). Mek1 kinase subsequently phosphorylates Ndt80 to diminish its DNA binding activity thereby ensuring that Ndt80 target genes, some of which promote JM resolution and progression beyond the pachytene stage, remain inactive until most DNA breaks are adequately processed (Chen *et al.* 2018). The recombination checkpoint also triggers Swe1 kinase activity, which indirectly stabilizes the Sum1 repressor complex via inhibitory phosphorylation of CDK (Shin *et al.* 2010). Finally, nuclear localization of Ndt80 is regulated by the recombination checkpoint, indicating that Ndt80 activity is also controlled by a spatial redistribution mechanism (Wang *et al.* 2011).

### Meiotic commitment

When yeast cells in prophase I are shifted to rich growth medium, meiosis is aborted and diploid cells return to vegetative growth. Cells accomplish this “return to growth” process by rapidly degrading meiotic chromosomal structures and repairing recombination intermediates with minimal crossover formation (Zenvirth *et al.* 1997; Dayani *et al.* 2011). However, following Ndt80 activation and a step known as commitment, cells will complete meiosis regardless of a change in external cues [reviewed in (Winter 2012)]. Interestingly, commitment is extremely sensitive to Ndt80 dosage: When the abundance of *NDT80* transcript is cut in half, the meiotic commitment point shifts such that even cells undergoing the meiosis I division will exit the meiotic program and initiate mitotic cell cycling (Tsuchiya *et al.* 2014). CDK kinase Ime2, polo-like kinase Cdc5, and 14-3-3 proteins Bmh1 and Bmh2 are critical for establishing and/or maintaining meiotic commitment (Gavade *et al.* 2022). Meiotic commitment furthermore relies on the combined action of the Rad53-mediated DNA damage checkpoint and the Bub2-mediated spindle position checkpoint pathways (Ballew and Lacefield 2019).

## Metaphase I

**Fig. 7.**
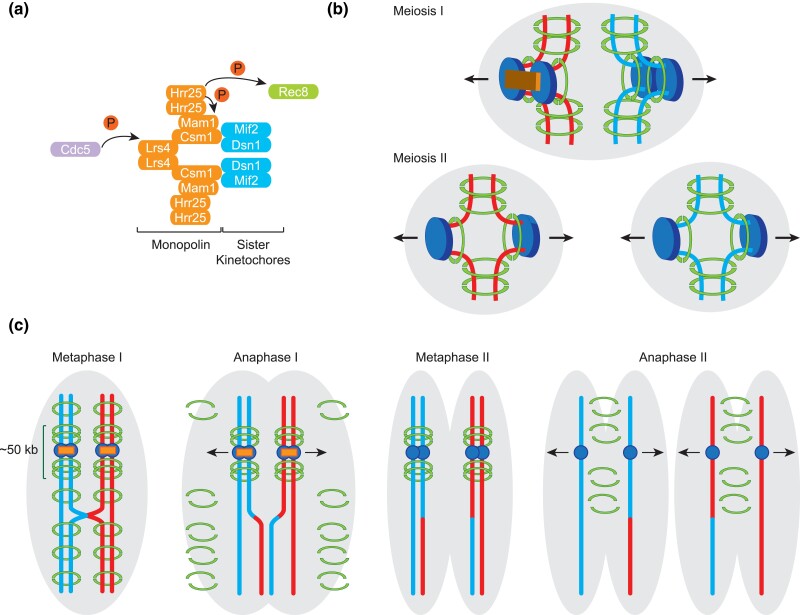
Chromosome disjunction during meiosis I and II. a) Following prophase I exit, the monopolin complex (Mam1, Lrs4, Csm1, Hrr25) is assembled at kinetochores to regulate the attachment of kinetochores from both sister chromatids to the same microtubule of the meiosis I spindle. b) Monopolin (depicted as a brown bar) fuses sister kinetochores during meiosis I to ensure monopolar attachment. Monopolin dissociates prior to meiosis II to allow bipolar attachment of sister kinetochores as also seen in mitosis. c) Stepwise loss of cohesion. At the metaphase I/anaphase I transition, cohesin is specifically cleaved along the chromosome arms resulting in dissolution of chiasmata and the disjunction of homologous chromosomes. Cohesin complexes in the pericentromeric regions (∼25 kb to either side of the centromere) are protected from cleavage by separase and provide sister chromatid cohesion during metaphase II. Loss of centromeric cohesion at anaphase II allows separation of sister chromatids.

Once cells have exited from prophase I, they initiate formation of the meiosis I spindle (Shirk *et al.* 2011; Kim *et al.* 2013; Newnham *et al.* 2013) and co-orient sister kinetochores to ensure that sister chromatids attach to the same spindle pole (Fig. 7, a–c). Monopolar attachment results from the meiosis I-specific fusion of sister kinetochores, such that the two kinetochores together present one attachment site for a single microtubule (Winey *et al.* 2005; Sarangapani *et al.* 2014). Fusion of sister kinetochores is brought about by monopolin, a protein complex composed of the meiosis-specific protein Mam1, the nucleolar factors Csm1 and Lrs4, and casein kinase Hrr25 (CK1δ/ε) (Fig. 7a) (Toth *et al.* 2000; Rabitsch *et al.* 2003; Petronczki *et al.* 2006). Upon prophase I exit, Csm1 and Lrs4 leave the nucleolus and join Mam1, Hrr25, condensin, and Zip1 at kinetochores (Rabitsch *et al.* 2003; Brito *et al.* 2010; Prajapati *et al.* 2018). Csm1 and Lrs4 form a heterotetrameric V-like structure that directly binds kinetochore components and is thought to physically clamp together sister kinetochores (Monje-Casas *et al.* 2007; Corbett *et al.* 2010; Corbett and Harrison 2012). Monopolin relocalization to kinetochores depends on the concerted action of DDK, Cdc5 (Lee and Amon 2003; Valentin *et al.* 2006; Monje-Casas *et al.* 2007; Lo *et al.* 2008; Matos *et al.* 2008), and the kinetochore-specific Cdc5-targeting factor “meikin” (meiosis-specific kinetochore factor) Spo13 (Klapholz and Esposito 1980; Katis, Matos, *et al.* 2004; Lee *et al.* 2004; Matos *et al.* 2008).

Even with co-oriented kinetochores, accurate meiosis I chromosome segregation requires that each sister pair only attaches to microtubules from one spindle pole and that the sister-pairs of the homologous partner chromosome attach to opposite poles (Fig. 7b) (Marston 2014). As in mitotic cells, proper orientation is continuously probed by the formation of kinetochore-microtubules attachments and their subsequent severing induced by Ipl1 (Aurora B) kinase (Monje-Casas *et al.* 2007; Meyer *et al.* 2013, 2021; Cairo *et al.* 2020). When bipolar attachment is achieved, spindle forces are resisted by crossovers together with sister chromatid cohesion along chromosome arms that link recombinant homologous chromosomes (Buonomo *et al.* 2000). The resulting tension physically pulls kinetochores away from the central spindle where Aurora B is localized, thereby stabilizing microtubule attachments (Liu *et al.* 2009). The accuracy of this process strongly relies on the distance of crossover sites from centromeres. Homologous chromosomes whose only crossovers are positioned close to the telomere are more likely to missegregate (Ross *et al.* 1996), whereas chromosomes that fail to form a chiasma (the physical manifestation of an interhomolog crossover event) within ∼180 kb of the centromere additionally require the spindle assembly checkpoint component Mad2 for faithful bipolar attachment (Shonn *et al.* 2003; Lacefield and Murray 2007).

Chromosomes that fail to form a crossover altogether (E0 or NEC—non-exchange chromosomes) present a particular problem for this system. Because even correct bipolar attachment does not lead to cohesion-dependent tension between NEC pairs, additional backup mechanisms are necessary to promote their segregation (Dawson *et al.* 1986). These mechanisms include the spindle assembly checkpoint as well as Zip1-dependent centromere associations. The spindle assembly checkpoint may provide additional time for proper NEC alignment (Shonn *et al.* 2003; Cheslock *et al.* 2005; Newnham *et al.* 2010). At the same time, the spindle checkpoint in yeast is quite weak in preventing the meiosis I division, as Mad2 delays meiosis I in mutants that entirely lack any crossover linkage between homologs (e.g. *spo11*) by only ∼2 hours after which meiotic progression occurs even without bipolar homolog attachment (Shonn *et al.* 2000). Persisting Zip1 during metaphase I provides physical connections between homologous centromeres, substituting for the lack of chiasmata, in contrast to Zip1-mediated leptotene-coupling which involves homology-independent associations. Zip1-dependent linkages by physically connecting centromeres at metaphase I (Loidl *et al.* 1994; Guerra and Kaback 1999; Kemp *et al.* 2004; Tsubouchi and Roeder 2005; Gladstone *et al.* 2009; Bardhan *et al.* 2010; Newnham *et al*. 2010; Kurdzo *et al.* 2018; Previato De Almeida *et al.* 2019). Yet, the system of distributive segregation begins to break down once more than two NECs are present (Dawson *et al.* 1986).

## Metaphase I to anaphase I transition

Anaphase I initiates once all homologous chromosome pairs have formed bipolar attachments. As in mitosis, stable bipolar attachment is monitored by the spindle assembly checkpoint, which detects unattached kinetochores and blocks the APC/C by inhibiting its activator Cdc20 (Shonn *et al.* 2000, 2002, 2003; Tsuchiya *et al.* 2011). Following kinetochore attachment, Cdc20 is free to associate with the APC/C to form a multi-subunit E3 ubiquitin ligase, which targets the anaphase inhibitor securin (Pds1) for proteasome-mediated degradation (Salah and Nasmyth 2000; Shonn *et al.* 2000; Oelschlaegel *et al.* 2005; Penkner *et al.* 2005). Pds1 is an inhibitory chaperone for separase Esp1, and its destruction allows separase to eliminate cohesin along chromosome arms through proteolytic cleavage of its kleisin subunit Rec8 (Buonomo *et al.* 2000). Esp1 cleaves Rec8 at two redundant sites within the protein, which leads to cohesin's dissociation and homolog separation (Klein *et al.* 1999; Buonomo *et al.* 2000). Efficient Rec8 cleavage requires its phosphorylation by multiple kinases, including DDK, CK1δ/ε, and Cdc5 (Brar *et al.* 2006; Petronczki *et al.* 2006; Matos *et al.* 2008; Katis *et al.* 2010). The activity of these kinases is constrained to the metaphase/anaphase transition by meikin Spo13, which counters the activity of cohesin kinases (Galander *et al.* 2019).

Whereas cohesins along chromosome arms are cleaved and/or removed prior to anaphase I, centromeric cohesion is maintained through meiosis I to allow proper tension-mediated alignment of sister chromatids in metaphase II (Fig. 7c). Overall cohesin binding is enriched around centromeres (Glynn *et al.* 2004) and establishment of stable centromeric cohesion requires the helicase Chl1 and a specialized replication factor C complex (RF-C Ctf18/Dcc1/Ctf8) (Petronczki *et al.* 2004). As cells enter anaphase I, centromeric cohesin is protected from separase-mediated cleavage by the shugoshin protein Sgo1 (Klein *et al.* 1999; Shonn *et al.* 2002; Katis, Galova, *et al.* 2004; Katis, Matos, *et al.* 2004; Kitajima *et al.* 2004; Marston *et al.* 2004; Kiburz *et al.* 2005). Sgo1, together with Aurora kinase Ipl1, recruits two alternative forms of phosphatase PP2A that act in parallel to prevent cohesin cleavage: PP2A associated with its regulatory subunit Rts1 (PP2A-Rts1) dephosphorylates Rec8, thereby protecting it from separase cleavage (Kitajima *et al.* 2006; Riedel *et al.* 2006; Tang *et al.* 2006; Yu and Koshland 2007; Xu *et al.* 2009), whereas PP2A-Cdc55 counters separase activation (Clift *et al.* 2009). Together, these mechanisms ensure that centromeric cohesion persists until metaphase II.

## Transitioning from meiosis I to meiosis II

The transition from meiosis I to meiosis II requires a transient drop in CDK activity to drive telophase I spindle disassembly and spindle pole body reduplication (Buonomo *et al.* 2003; Marston *et al.* 2003; Carlile and Amon 2008). A reduction in CDK activity is mediated by the temporary release of Cdc14 phosphatase from the nucleolus in anaphase I triggered by the FEAR signaling network (Kamieniecki *et al.* 2000; Buonomo *et al.* 2003; Marston *et al.* 2003; Sullivan *et al.* 2008). Once the spindle is disassembled, Cdc14 returns to the nucleolus and a rise in CDK activity (Cdc28 in association with cyclins Clb1 and Clb4) initiates the assembly of the two meiosis II spindles (Dahmann and Futcher 1995; Buonomo *et al.* 2003; Marston *et al.* 2003; Monje-Casas *et al.* 2007). Cells with an inactive FEAR network or hyperactive CDK fail to segregate their nucleolus and do not complete the meiotic spindle cycle. Instead, they undergo two rounds of chromosome segregation on a single spindle (Buonomo *et al.* 2003; Marston *et al.* 2003; Fuchs and Loidl 2004; Kerr *et al.* 2011), leading to the formation of diploid spores that exhibit a mix of reductional and equational segregation (Klapholz and Esposito 1980; Sharon and Simchen 1990; Hugerat and Simchen 1993; Kamieniecki *et al.* 2000; Zeng and Saunders 2000; Pfiz *et al.* 2002).

In mitotic cells, the telophase drop in CDK activity relicenses replication origins for another round of replication (Diffley 2010). During the meiosis I-to-meiosis II transition, this relicensing is prevented by the persistent activity of Ime2 (Benjamin *et al.* 2003; Phizicky *et al.* 2018), whose target sites are largely resistant to dephosphorylation by Cdc14 (Holt *et al.* 2007). Accordingly, deregulated Ime2 exhibits synthetic phenotypes with FEAR network mutants (Schindler and Winter 2006). Nevertheless, mutants that replicate their DNA between meiosis I and meiosis II have not been identified, although deregulation of CDK can cause multiple rounds of replication during prophase I (Strich *et al.* 2004; Rice *et al.* 2005; Sawarynski *et al.* 2009).

At the end of anaphase I, Spo13 and Mam1 are degraded, and Csm1 and Lrs4 return to the nucleolus, preparing chromosomes for meiosis II (Rabitsch *et al.* 2003; Katis, Matos, *et al.* 2004; Sullivan and Morgan 2007; Matos *et al.* 2008). The resulting loss of kinetochore mono-orientation allows Sgo1 along with Ipl1 to promote the bipolar attachment of sister kinetochores on the metaphase II spindle (Monje-Casas *et al.* 2007; Kiburz *et al.* 2008; Nerusheva *et al.* 2014). In addition, any remaining DSBs are repaired (Cartagena-Lirola *et al.* 2008) and leftover dHJs are removed by meiosis-II-specific activation of the Yen1 resolvase (Matos *et al.* 2011). Finally, anaphase II is triggered by a second round of Esp1 activation, Sgo1 degradation and pericentromeric Rec8 cleavage, which is sufficient to separate sister chromatids and yield the four haploid products of meiosis (Buonomo *et al.* 2000; Salah and Nasmyth 2000; Mengoli *et al.* 2021).

## Non-chromosomal genetic elements in meiosis

Although the meiotic program is primarily geared toward ensuring the faithful inheritance of chromosomal DNA, meiosis is also a time of extraordinary activity for extra-chromosomal and extra-nuclear genetic elements. Mitochondria undergo a series of gross morphological changes during prophase I (Miyakawa *et al.* 1984; Gorsich and Shaw 2004) and ultimately detach from the cell cortex in an Ndt80-dependent manner to associate with meiotic nuclei during meiosis I and II (Miyakawa *et al.* 1984; Sawyer *et al.* 2019). This nuclear attachment is thought to promote mitochondrial inheritance (Gorsich and Shaw 2004; Suda *et al.* 2007), although only about 50% of mitochondrial genomes are ultimately packaged into spores (Brewer and Fangman 1980). Intriguingly, parasitic M and L double-stranded RNAs, which exist as virus-like particles in the cytoplasm, use poorly understood mechanisms to also promote their packaging into spores (Brewer and Fangman 1980). The ultimate abundance of these RNAs is constrained by Nuc1 endonuclease, which is released from mitochondria upon Ndt80 activation (Gao *et al.* 2019) and also degrades nuclei that failed to become encapsulated into spores (Eastwood *et al.* 2012). At the same time, retrotransposon RNAs become highly expressed at the end of prophase I by taking advantage of Ndt80-dependent regulation (Laureau *et al.* 2021), whereas aging-associated extra-chromosomal rDNA circles are preferentially eliminated (Unal *et al.* 2011; King *et al.* 2019). Meiosis, therefore, is a time of major reorganization of non-chromosomal and mobile genetic elements.

## Outlook

Budding yeast was established as a model organism for meiosis in the 1970s, and over 3,000 papers have since reported findings using this system. Yeast has provided increasingly detailed insights into the temporal and functional relationship between the molecular events of DNA metabolism and chromosome morphogenesis at the microscopic level. Studies in other organisms have demonstrated that the processes of meiosis and the meiotic machinery are evolutionarily conserved in higher eukaryotes. At the same time, many questions remain unresolved: How do homologous chromosomes identify each other during the pairing process? How are frequency and outcome of recombination events in different chromosome regions controlled? How are different pathways of meiotic DSB processing coordinated? And how are chromosomal events coordinated with the pathways operating under the control of the cytoplasmic machinery? Many of these open questions revolve around interactions between players that lack physical contacts, between chromosome loops and axes, between different regions along the same chromosome, and between different cellular compartments. These interactions imply signaling processes that remain to be discovered. Furthermore, many of the molecular pathways identified in meiosis involve components also expressed in vegetative cells, raising the possibility that the same processes also integrate mitotic cellular function. Future work may show that processes in DNA metabolism and chromosome morphogenesis previously thought to be limited to meiosis have equivalents in mitotically dividing cells (Kleckner *et al.* 2004). The experience from the first 50 years of molecular meiosis research suggests that studies in budding yeast could well be at the forefront of these discoveries.
